# A FRET probe with AIEgen as the energy quencher: dual signal turn-on for self-validated caspase detection†

**DOI:** 10.1039/c6sc00055j

**Published:** 2016-03-16

**Authors:** Youyong Yuan, Ruoyu Zhang, Xiamin Cheng, Shidang Xu, Bin Liu

**Affiliations:** a Department of Chemical and Biomolecular Engineering, National University of Singapore 4 Engineering Drive 4 Singapore 117585 cheliub@nus.edu.sg; b Institute of Materials Research and Engineering, Agency for Science, Technology and Research (A*STAR) 3 Research Link 117602 Singapore

## Abstract

The accurate detection of biological substances is highly desirable to study various biological processes and evaluate disease progression. Herein, we report a self-validated fluorescent probe which is composed of a coumarin fluorophore as the energy donor and a fluorogen with aggregation-induced emission characteristics (AIEgen) as the energy quencher linked through a caspase-3 specific peptide substrate. Unlike the traditionally widely studied fluorescence resonance energy transfer (FRET) probes, our new generation of FRET probe is non-fluorescent itself due to the energy transfer as well as the dissipation of the acceptor energy through the free molecular motion of AIEgen. Upon interaction with caspase-3, the probe displays strong green and red fluorescent signals synchronously due to the separation of the donor–quencher and aggregation of the released AIEgen. The fluorescence turn-on with dual signal amplification allows real-time and self-validated enzyme detection with a high signal-to-background ratio, providing a good opportunity to accurately monitor various biological processes in a real-time manner.

## Abstract 2

A new generation of FRET probe, based on coumarin (Cou) as the energy donor and AIEgen (TPETP) as the energy quencher conjugated *via* an Asp-Glu-Val-Asp (DEVD) substrate was developed for self-validated caspase-3 detection with dual fluorescent signal turn-on.

## Graphical abstract

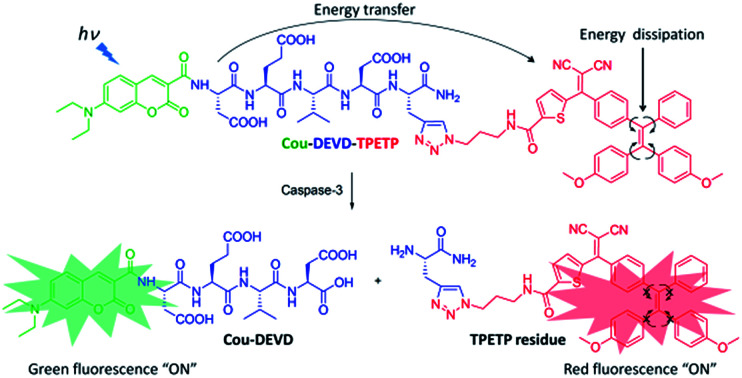

## Introduction

Fluorescent probes have attracted increasing attention in biomedical research due to their high sensitivity, good selectivity, non-invasiveness and the capability of real-time detection.^1^ Fluorescence turn-on probes are superior to turn-off probes due to their lower background signal and higher signal output.^2^ Fluorescence resonance energy transfer (FRET) is one of the most widely exploited mechanisms for the design of fluorescence turn-on probes, which have been successfully utilized in sensing, imaging, environmental monitoring, and medical diagnosis.^3^ Two strategies have been generally utilized to design FRET probes. One is to conjugate a fluorescent dye with a quencher (donor–quencher model) and the other is to link two fluorescent dyes which can form the donor–acceptor pair (donor–acceptor model).^4^ Once these FRET probes are exposed to analytes, the separation between the donor and quencher/acceptor leads to readable signals. While the donor–quencher model exhibits a single fluorescent signal output, the donor–acceptor model shows donor fluorescence turn-on at the expense of the acceptor emission. The single fluorescent signal turn-on can only provide limited information, which is sometimes insufficient for accurate detection.^5^ Recently, several “always on” probes with different image modalities have been developed for self-validated bioimaging in order to provide more accurate results.^5^ Up to now, a variety of biological probes based on fluorescence imaging, magnetic resonance imaging (MRI), positron emission tomography (PET) and photoacoustic imaging have been actively explored to offer dual signal output in one study.^6^ However, single fluorescence turn-on probes with a dual signal output upon encountering a specific analyte have not been reported yet. It is thus of great interest to develop single fluorescent probes with dual signal turn-on at different emission wavelengths for self-validated sensing and imaging, which remains challenging.

In recent years, fluorogens with aggregation-induced emission characteristics (AIEgens) have attracted considerable attention in biosensing and bioimaging.^7^ Opposite to the traditional fluorophores that show a notorious phenomenon known as aggregation-caused quenching (ACQ),^8^ AIEgens are non-emissive in a molecularly dissolved state but can be induced to emit strongly in aggregates due to the restriction of intramolecular motion (RIM) and prohibition of energy dissipation *via* non-radiative channels.^9^ Based on this unique property, several fluorescence turn-on probes have been developed for the detection and imaging of various analytes.^7*c*^ These probes are generally based on AIEgens conjugated to hydrophilic recognition elements. When the AIE probes are well-dissolved in aqueous media, the background fluorescence is very low. Upon specific analyte recognition, the probes are cleaved to release the hydrophobic AIEgens and yield a bright fluorescence. As compared to traditional FRET probes which require dual labelling to realize fluorescence turn-on, these singly labelled AIE probes show high simplicity, which makes them very useful in the development of multifunctional probes for the monitoring of multiple processes in one go.^7*c*^ These successful examples have motivated us to explore whether the energy dissipation of AIEgens could affect other fluorophores conjugated to the probe. In this case, it would offer a new generation of energy quenchers which are able to fluoresce once they are separated from the energy donor and offer for the first time a dual signal turn-on probe for self-validated biosensing and bioimaging.

**Scheme 1:**
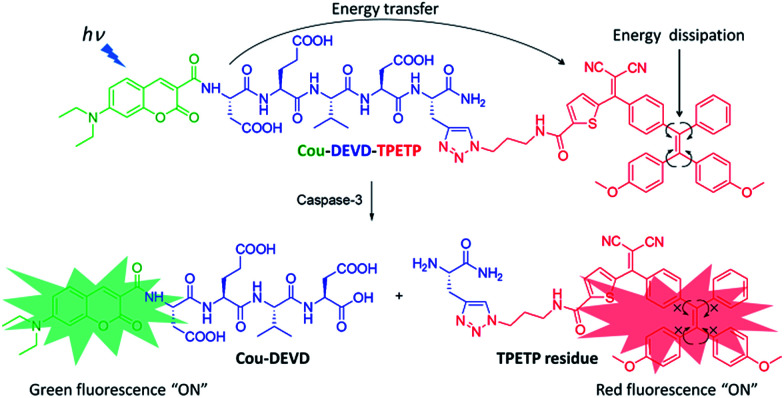
Schematic illustration of the FRET probe using AIEgen as an energy quencher with dual signal output for self-validated caspase-3 detection.

As a proof of concept, in this contribution we report a fluorescent probe based on coumarin (Cou) as the energy donor and AIEgen as the energy quencher conjugated *via* an Asp-Glu-Val-Asp (DEVD) substrate for caspase-3 detection (Scheme 1).^10^ The probe itself is non-fluorescent due to the energy transfer and dissipation of the acceptor energy through the free motion of AIEgens. Upon addition of caspase-3, it displays a strong green fluorescence of Cou due to the separation of the donor–acceptor and an intense red fluorescence of AIEgen due to the aggregation of the released AIEgen residue. This dual fluorescent signal turn-on can be used for caspase-3 detection both in solution and in cells, which opens new avenues for the development of next generation self-validated FRET probes with high signal-to-background ratios and fluorescence amplifications.

## Results and discussion

**Scheme 2:**
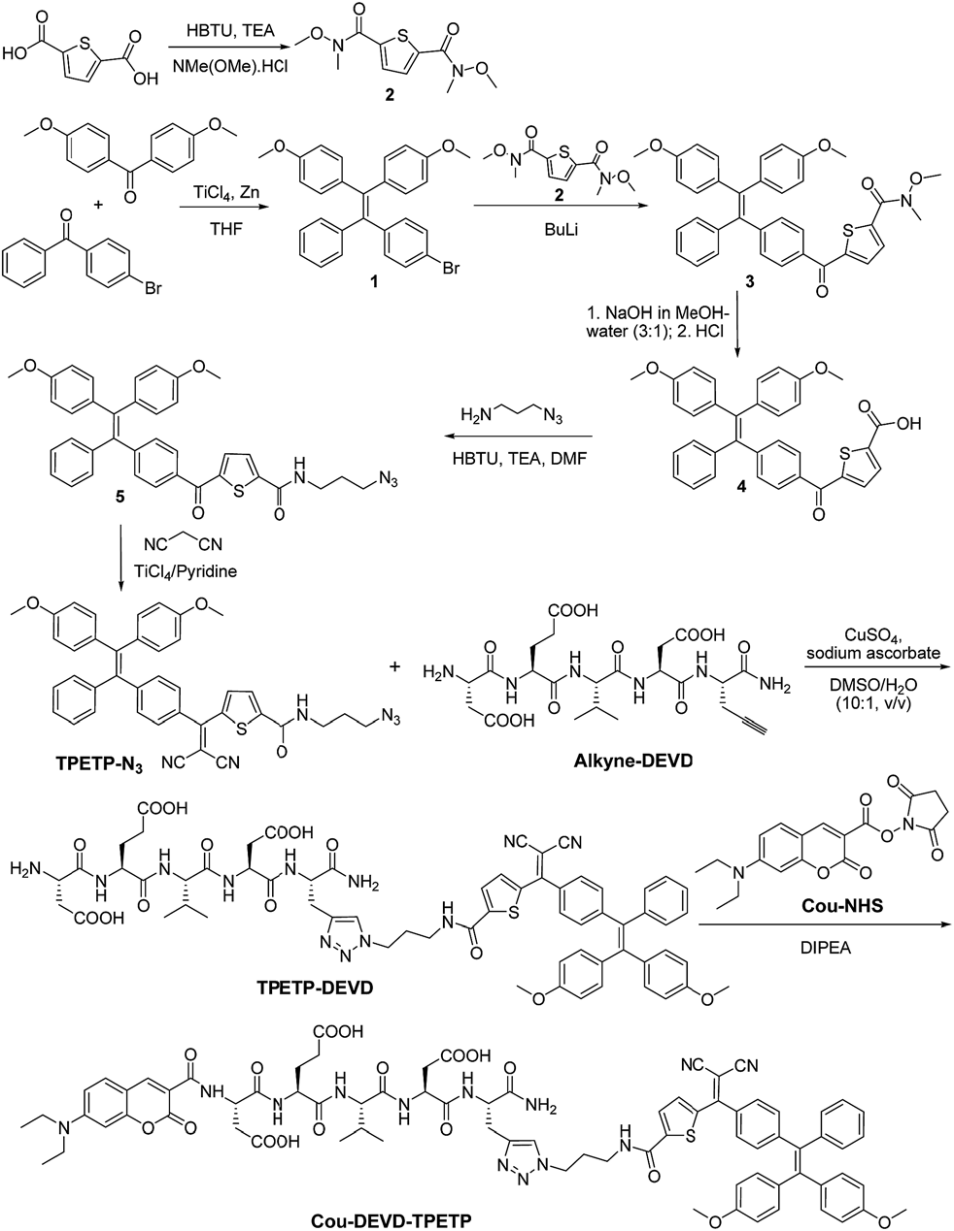
Synthetic route to the probe Cou–DEVD–TPETP. HBTU: *O*-(benzotriazol-1-yl)-*N*,*N*,*N*′,*N*′-tetramethyluronium hexafluorophosphate; TEA: triethylamine; TiCl_4_: titanium(iv) chloride; Zn: zinc; THF: tetrahydrofuran; BuLi: butyllithium; DMF: *N*,*N*-dimethylformamide; DIPEA: *N*,*N*-diisopropylethylamine; Cou-NHS: 7-(diethylamino)coumarin-3-carboxylic acid *N*-succinimidyl ester.

Recently, some AIEgens with red emission have been developed for bioimaging and tetraphenylethenethiophene (TPETP) was selected for this study.^11^ The synthetic route to azide-functionalized TPETP (TPETP-N_3_) and the probe Cou–DEVD–TPETP are shown in Scheme 2. Compound 1 was synthesized by McMurry reaction between bis(4-methoxyphenyl)methanone and (4-bromophenyl)(phenyl)methanone in the presence of TiCl_4_ and Zn. Subsequently, compound 1 was reacted with compound 2 in the presence of butyllithium to yield compound 3. The carboxyl group of compound 3 was deprotected by sodium hydroxide in a methanol–water mixture, which was further reacted with 3-azidopropan-1-amine to yield compound 5. Compound 5 was further reacted with malononitrile on a SiO_2_ support to produce azide-functionalized TPETP (TPETP-N_3_). The “click” reaction between TPETP-N_3_ and alkyne-functionalized DEVD catalyzed by copper(ii) sulfate (CuSO_4_) and sodium ascorbate (Na Asc) afforded amine terminated TPETP–DEVD, which was further reacted with 7-(diethylamino)coumarin-3-carboxylic acid *N*-succinimidyl ester (Cou-NHS) in the presence of *N*,*N*-diisopropylethylamine (DIPEA) to afford Cou–DEVD–TPETP in a 52% yield after HPLC purification. Detailed characterization data are shown in Fig. S1–S8.† The AIE property of TPETP-N_3_ was confirmed by studying its PL spectra in DMSO or DMSO/water mixtures (v/v = 1/99). As shown in Fig. S9A,† TPETP-N_3_ is almost non-fluorescent in DMSO which should be due to the easy intramolecular motions of the TPE phenyl rings in benign solvents.^7*a*^ However, TPETP-N_3_ in DMSO/water mixtures (v/v = 1/99) is highly fluorescent, and shows a 110-fold brighter fluorescence than that in DMSO. The increase in fluorescence intensity is attributed to the aggregation of TPETP-N_3_, which restricts the intramolecular motion when the water fraction is increased. The formation of TPETP-N_3_ aggregates in aqueous media was also confirmed by laser light scattering (LLS) measurements and transmission electron microscopy (TEM) imaging (Fig. S9†), while there is no LLS signal detected in DMSO solution. The absorption and emission spectra of Cou and TPETP-N_3_ are shown in Fig. S10.† The emission of Cou and the absorption of TPETP-N_3_ show a perfect spectral overlap, indicating that they could form an energy transfer pair.

**Fig. 1:**
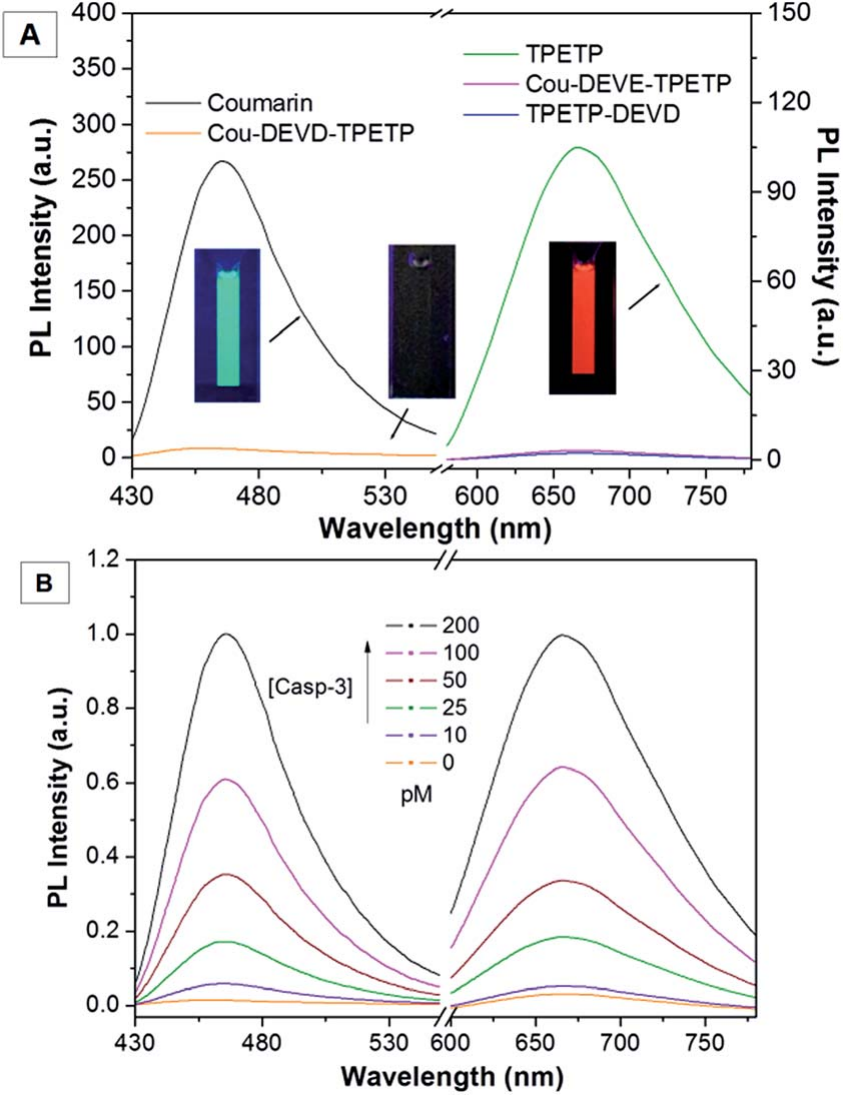
(A) Photoluminescence (PL) spectra of Cou, TPETP-N_3_ and Cou–DEVD–TPETP (10 μM) in DMSO/PBS buffer (v/v = 1/99). (B) PL spectra of Cou–DEVD–TPETP (10 μM) upon incubation with different concentrations of caspase-3 (*λ*_ex_: 405 nm, emission collected from 430–550 nm is from Cou–DEVD and that at 600–780 nm is from the TPETP residue).

To validate the energy transfer between Cou and TPETP-N_3_, the optical properties of Cou, TPETP-N_3_ and Cou–DEVD–TPETP were studied in DMSO/PBS buffer (v/v = 1/99). Cou and TPETP-N_3_ have absorption maxima at 445 and 470 nm, with emission maxima at 465 and 665 nm, respectively (Fig. S10†). As both Cou and TPETP-N_3_ show an obvious absorption at 405 nm, this offers the possibility of dual signal collection upon a single wavelength excitation. As shown in Fig. 1A, upon excitation at 405 nm, both Cou and TPETP-N_3_ are highly fluorescent while Cou–DEVD–TPETP emits very weakly, which indicates that the probe has an extremely low background signal, as designed. The rationale behind the fluorescence quenching of the Cou and TPETP parts in the probe is due to the energy transfer from the former to the latter, and the dissipation of the AIEgen energy through the free motion of the molecules. The fluorescence of the probe remains weak in aqueous media with different ionic strengths or in a cell culture medium (Fig. S11†). Upon addition of caspase-3, the cleavage of DEVD separates Cou–DEVD away from the proximity of TPETP. This leads to the recovery of the green fluorescence of Cou and synchronously allows the formation of TPETP residue aggregates with a red fluorescence turn-on. As shown in Fig. 1B, upon treatment with caspase-3 at different concentrations for 1 h at 37 °C, the emissions of Cou–DEVD and the TPETP residue exhibit a concentration-dependent turn-on at 465 nm and 665 nm, respectively. When the probe was treated with 200 pM caspase-3 for 1 h, the fluorescence of Cou–DEVD and the TPETP residue showed a 55- and 37-fold increase compared to the intrinsic emission in the probe, respectively. In addition, the peak PL intensities of Cou and TPETP are shown in Fig. S12,† which correlate linearly to the concentration of caspase-3 with *R*^2^ = 0.97, indicating that the enzyme concentration can be quantified and self-validated through monitoring the PL intensity changes of Cou and TPETP. In addition, in the presence of the caspase-3 inhibitor 5-[(*S*)-(+)-2-(methoxymethyl)pyrrolidino]sulfonylisatin, the probe remains weakly emissive even after the treatment with caspase-3. A kinetic analysis of the enzymatic reaction was also carried out by incubating caspase-3 with different concentrations of Cou–DEVD–TPETP at 37 °C. As shown in Fig. S13,† the Michaelis constant (*K*_M_) and kinetic constant (*k*_cat_) were calculated to be 7.70 μM and 2.69 s^−1^, which are comparable to the previous FRET probe.^10*a*^ The specific cleavage of DEVD by caspase-3 was further confirmed by a reverse phase HPLC and mass spectrometry study through the formation of Cou–DEVD and the TPETP residue (Fig. S14†).

**Fig. 2:**
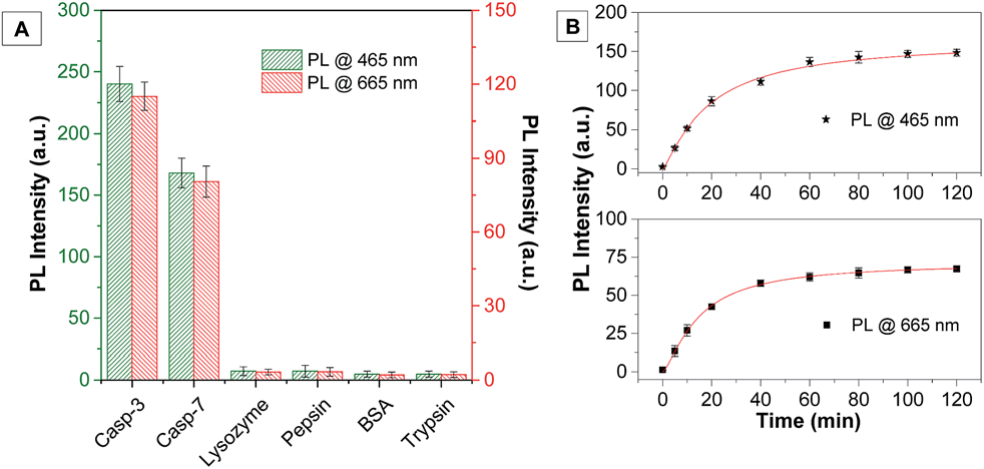
(A) PL intensities monitored at both 465 and 665 nm for Cou–DEVD–TPETP (10 μM) upon treatment with various proteins; (B) time-dependent PL intensities for Cou–DEVD–TPETP (10 μM) upon addition of caspase-3 (*λ*_ex_: 405 nm).

The selectivity of the probe was studied by incubating the probe with different caspase enzymes as well as several proteins including lysozyme, pepsin, bovine serum albumin and trypsin. As shown in Fig. 2A and S15A,† only the probe treated with caspase-3/-7 displays a significant fluorescence increase, confirming the specificity of the probe for caspase-3/-7. The time-dependent fluorescence change of the probe after incubation with caspase-3/-7 and a cell lysate of normal and apoptotic cells was also studied. As shown in Fig. 2B, a quick and steady fluorescence increase at both 465 and 665 nm is observed when caspase-3 is added. On the other hand, the HeLa cells were induced to undergo apoptosis by the treatment of staurosporine (STS), a commonly used apoptosis inducer,^12^ and the cell lysates were incubated with the probe. As shown in Fig. S15,† the fluorescent signals at both wavelengths increase along with incubation time which is similar to the solution study shown in Fig. 2B. In contrast, no fluorescence change is observed when the probe is incubated with the HeLa cell lysate without STS treatment, indicating that the probe is stable with cellular proteins and it can be specifically recognized by the caspase enzyme with self-validation.

**Fig. 3:**
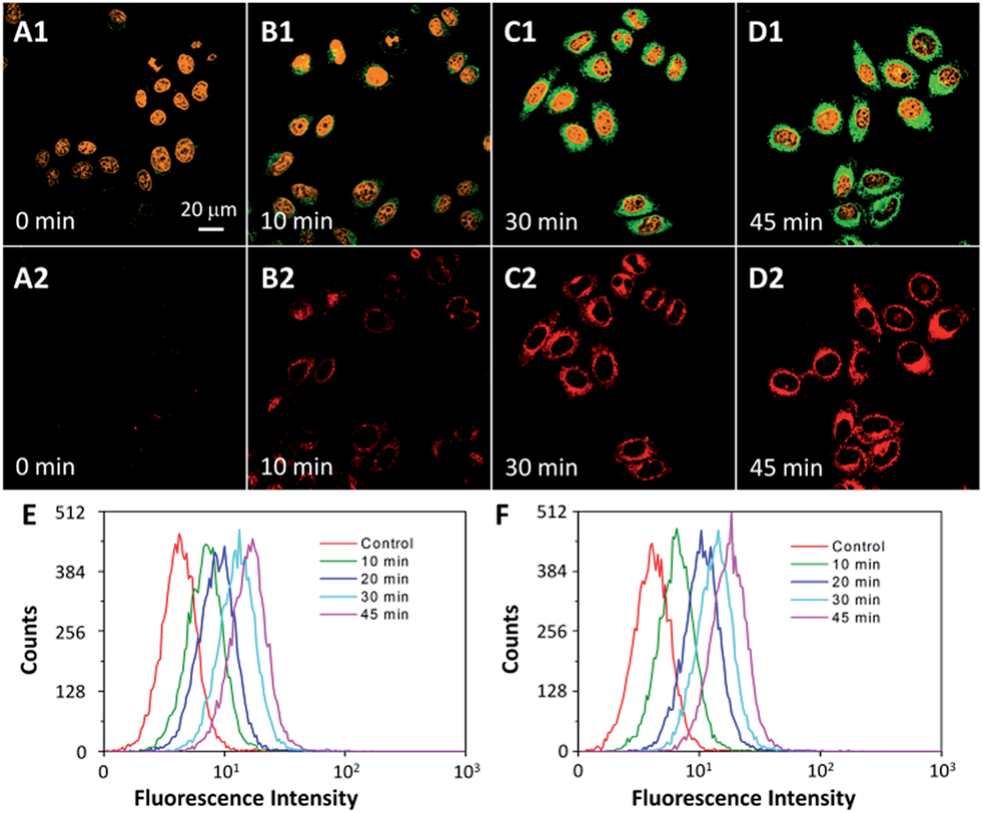
Confocal images of Cou–DEVD–TPETP (10 μM) incubated HeLa cells upon treatment with STS (1 μM) for different times. Green fluorescence (Cou–DEVD, *E*_x_: 405 nm; *E*_m_: 505–525 nm); orange fluorescence (nucleus dyed with SYTO® orange, *E*_x_: 543 nm, *E*_m_: 610–640 nm); A1–D1 are the overlay images of the fluorescence of Cou and SYTO® orange; red fluorescence (TPETP residue, A2–D2, *E*_x_: 405 nm, *E*_m_: >650 nm). (E and F) Flow cytometric analysis of Cou–DEVD (E) and the TPETP residue (F) fluorescence in HeLa cells after treatment with STS (1 μM).

To explore the potential of using Cou–DEVD–TPETP for caspase imaging in live cells, the probe was incubated with HeLa and MDA-MB-231 cells and subsequently treated with STS. The fluorescence changes at both wavelengths were monitored by confocal laser scanning microscopy. As shown in Fig. 3 and S16,† the fluorescent signals of Cou–DEVD and the TPETP residue increase gradually and synchronously with the cellular apoptotic progress upon addition of STS. The fluorescence changes after STS treatment were also confirmed by flow cytometric analysis (Fig. 3E and F). These results clearly support the idea that the probe with dual signal turn-on can be used for the self-validation of caspase-3 activation and for real-time monitoring of the apoptosis process in live cells.

**Fig. 4:**
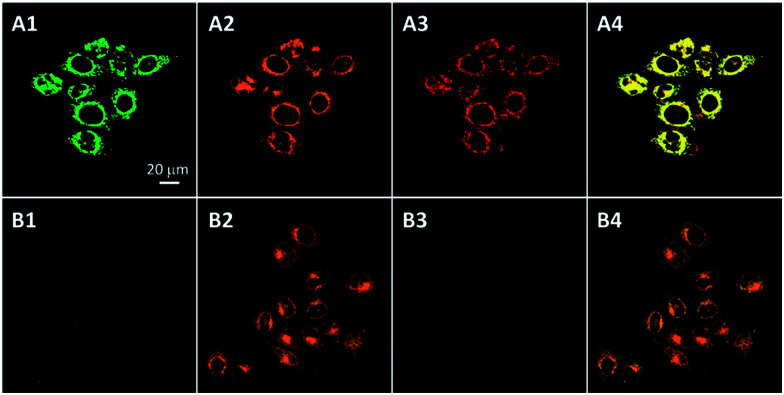
Confocal images of apoptotic HeLa cells treated with Cou–DEVD–TPETP (10 μM) in the absence (A) and presence (B) of the caspase-3 inhibitor and stained with an anti-caspase-3 primary antibody and a Texas Red-labeled secondary antibody. Green fluorescence (Cou–DEVD, A1, B1, *E*_x_: 405 nm; *E*_m_: 505–525 nm); orange fluorescence (Texas Red, A2, B2, *E*_x_: 543 nm, *E*_m_: 610–640 nm); red fluorescence (TPETP residue, A3, B3, *E*_x_: 405 nm, *E*_m_: >650 nm); A4 and B4 are the overlay images of A1–A3 and B1–B3, respectively. Due to the low absorbance of TPETP at 543 nm, its emission spectral overlap with Texas Red is negligible.

To further confirm that the probe can image cell apoptosis, HeLa cells were co-stained with an anti-caspase-3 primary antibody and a Texas Red-labeled secondary antibody. As shown in Fig. 4A, the fluorescence of Cou–DEVD and the TPETP residue in HeLa cells with STS treatment overlaps well with the immunofluorescence signals from Texas Red. However, when the cells are pretreated with the caspase-3/-7 inhibitor, both intensities are greatly reduced while the fluorescence of Texas Red remains (Fig. 4B). This is because the activity of the caspase-3/-7 enzyme is prohibited upon inhibitor treatment, but the apoptosis process is not affected. Similar results were also observed in MDA-MB-231 cells (Fig. S17†). Overall, these results further confirm the caspase-3/-7 specific turn-on of the probe fluorescence in cells.

**Fig. 5:**
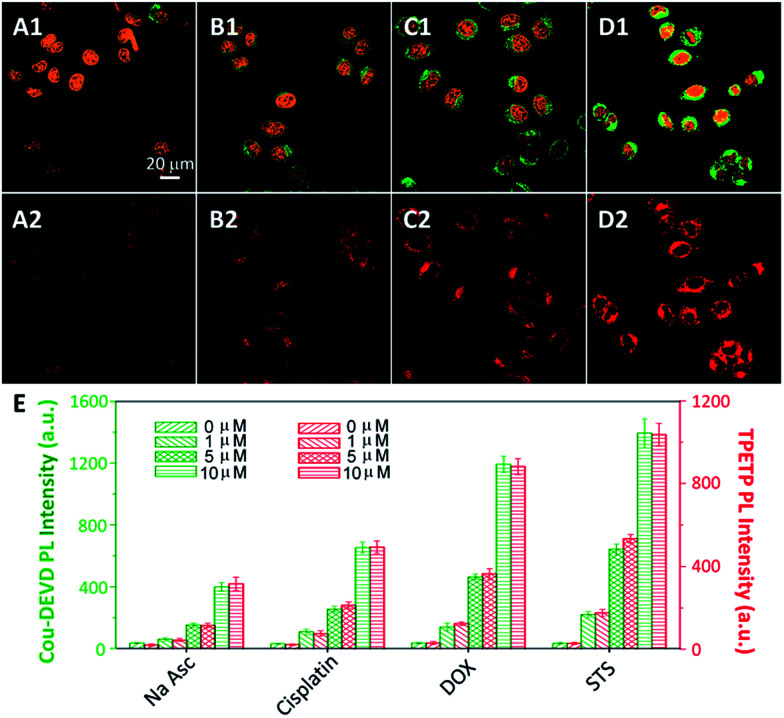
Confocal images of Cou–DEVD–TPETP (10 μM) incubated HeLa cells upon treatment with (A) sodium ascorbate (Na Asc), (B) cisplatin, (C) DOX and (D) STS. Green fluorescence (Cou–DEVD, *E*_x_: 405 nm; *E*_m_: 505–525 nm); orange fluorescence (nucleus dyed with SYTO® orange, *E*_x_: 543 nm, *E*_m_: 610–640 nm); A1–D1 are the overlay images of the fluorescence of Cou and SYTO® orange; red fluorescence (TPETP residue, A2–D2, *E*_x_: 405 nm, *E*_m_: >650 nm). (E) PL intensities of Cou–DEVD and the TPETP residue in HeLa cells treated with Na Asc, cisplatin, DOX and STS at different concentrations.

It is known that most drugs induce cell death through the apoptosis pathway.^13^ To verify the potential of this probe for self-validated drug screening, the probe incubated cells were treated with several cell apoptosis inducing drugs and the apoptosis-inducing capabilities were evaluated by monitoring the fluorescent signal changes. As shown in Fig. 5, the strongest fluorescence of Cou–DEVD and the TPETP residue is observed when the cells are treated with STS, while a moderate fluorescence is observed when the cells are treated with the anticancer drug doxorubicin (DOX) or cisplatin. The cells treated with Na Asc show the lowest fluorescence, indicating that Na Asc is not a good apoptosis inducer. Similar results were also observed in MDA-MB-231 cells (Fig. S18†), revealing the generality of the probe. To test whether the probe could be used to quantify the capability of different drugs in inducing cell apoptosis, the cells were further treated with different amounts of Na Asc, cisplatin, DOX and STS and the fluorescent signals were studied. As shown in Fig. 5C, the fluorescent signals of Cou–DEVD and the TPETP residue intensify synchronously with the drug concentration for all the four drugs. It should be noted that the probe (up to 25 μM) does not show any obvious cytotoxicity to both cells after 48 h incubation (Fig. S19†). Collectively, these results indicate that Cou–DEVD–TPETP can quantitatively analyze the capability of different drugs to induce cell apoptosis in living cells with self-validation, which offers a new opportunity to accurately evaluate the efficiency of new anticancer drugs.

## Conclusions

In summary, we developed a simple but unique fluorescent probe with a dual signal turn-on for accurate caspase-3 detection with self-validation. Thanks to the unique property of AIEgen, the fluorescence of the probe is initially quenched, but a two-signal turn-on is produced upon interaction with the caspase-3 enzyme. The dual-signal turn-on enables real-time monitoring of caspase-3 activity in solution and in live cells with a high efficiency, which has been utilized for self-validated enzyme detection and drug screening. Compared to traditional FRET probes that show a single fluorescence turn-on upon interaction with the analytes, the probe developed in this work using AIEgen as the energy quencher does not complicate the probe design, but offers for the first time a two-signal turn-on upon analyte recognition. In addition, this is the first time that the energy quencher could change its role to a signal reporter upon analyte recognition. Our design strategy for the AIEgen based FRET probe can be generalized to other probes simply by changing DEVD to another cleavable substrate, which will open new avenues for self-validated diagnosis, imaging and drug screening applications. For real practical applications, there is a need to evaluate the *in vivo* cytotoxicity and design probes with a longer absorption and emission wavelength.

## Supplementary Material

SC-007-C6SC00055J-s001
