# Supplementary material for: A FRET probe with AIEgen as the energy quencher: dual signal turn-on for self-validated caspase detection
Source: Chem Sci. 2016 Mar 16;7(7):4245–50. doi: 10.1039/c6sc00055j (PMC6013802; doi:10.1039/c6sc00055j)
Supplement: SC-007-C6SC00055J-s001 [file SC-007-C6SC00055J-s001.pdf]

Electronic Supplementary Material (ESI) for Chemical Science.
This journal is © The Royal Society of Chemistry 2016

Supporting Information

FRET Probe with AIEgen as the Energy Quencher: Dual Signal Turn-On for Self-Validated Caspase Detection

Youyong Yuan, Ruoyu Zhang, Xiamin Cheng, Shidang Xu, and Bin Liu*

Materials and Methods

Materials and characterization: Cisplatin, doxorubicin (DOX), trifluoroacetic acid (TFA), 7-(diethylamino)coumarin-3-carboxylic acid *N*-succinimidyl ester (Cou-NHS), *N*-(3-dimethylaminopropyl)-*N′*-ethylcarbodiimide hydrochloride (EDC), *N*-hydroxysuccinimide (NHS), *N*, *N*-diisopropylethylamine (DIPEA), triethylamine (TEA), *N,N,N′,N′*-tetramethyl-*O*-(1*H*-benzotriazol-1-yl)uronium hexafluorophosphate, *O*-(benzotriazol-1-yl)-*N,N,N′,N′*-tetramethyluronium hexafluorophosphate (HBTU), copper(II) sulfate ($CuSO_4$), sodium ascorbate, *N*, *N*-diisopropylethylamine (DIPEA), anhydrous dimethyl sulfoxide (DMSO), anhydrous *N*, *N*-dimethylformamide (DMF), 3-(4,5-dimethylthiazol-2-yl)-2,5-diphenyltetrazolium bromide (MTT) and other chemicals were all purchased from Sigma-Aldrich or Alfa Aesar and used as received without further purification. Tetrahydrofuran (THF) and dichloromethane were dried by distillation using sodium as drying agent and benzophenone as indicator. All non-aqueous reactions were carried out under nitrogen atmosphere in oven-dried glassware. Deuterated solvents with tetramethylsilane (TMS) as internal reference were purchased from Cambridge Isotope Laboratories Inc.. Alkyne-functionalized DEVD (Asp-Glu-Val-Asp) peptide was purchased from GL Biochem Ltd (Shanghai).

Dulbecco's Modified Essential Medium (DMEM) is a commercial product of Invitrogen. Milli-Q water was supplied by Milli-Q Plus System (Millipore Corporation, United States). Phosphate-buffer saline (PBS, 10×) buffer with pH = 7.4 is a commercial product of 1st BASE (Singapore). Milli-Q water (18.2 MΩ) was used to prepare the buffer solutions from the 10× PBS stock buffer. 1× PBS contains NaCl (137 mM), KCl (2.7 mM), Na_2HPO_4 (10 mM), and KH_2PO_4 (1.8 mM). Recombinant human caspase-3 was purchased from R&D Systems. Caspase-3/-7 inhibitor 5-[(*S*)-(+)-2-(methoxymethyl)pyrrolidino]sulfonylisatin was purchased from Calbiochem. Staurosporine (STS) was purchased from Biovision. Cleaved caspase-3 (Asp175) (5A1E) rabbit mAb (#9664) was purchased

from Cell Signaling. Mouse anti-rabbit IgG-TR (sc-3917) was purchased from Santa Cruz. SYTO® orange, fetal bovine serum (FBS) and trypsin-EDTA solution were purchased from Life Technologies.

NMR spectra were measured on a Bruker ARX 300/400/500 NMR spectrometer. Chemical shifts are reported in parts per million referenced with respect to residual solvent ($CDCl_3$ = 7.26 ppm and $(CD_3)_2SO$ = 2.50 ppm) for ^{1}H NMR and ($CDCl_3$ = 77.0 ppm and $(CD_3)_2SO$ = 40.0 ppm) for ^{13}C NMR. The extent of reaction was monitored by thin layer chromatography (TLC) using Merck 60 F254 pre-coated silica gel plates with fluorescent indicator UV254. After the plates were subjected to elution in the TLC chamber, the spots were visualized under UV light or using the appropriate stain (I_2, $KMnO_4$, ninhydrin or ceric ammonium molybdate (CAM)). Flash column chromatography was carried out using Merck silica gel (0.040-0.063). A 0.1% trifluoroacetic acid solution in H_2O and acetonitrile was used as the eluent for high-performance liquid chromatography (HPLC) experiments (Agilent). Mass spectra were recorded on Agilent 5975 DIP-MS for electron impact (EI) and the AmaZon X LC-MS for electrospray ionization (ESI). Particle size and size distribution were determined by laser light scattering (LLS) with a particle size analyzer (90 Plus, Brookhaven Instruments Co., United States) at a fixed angle of 90° at room temperature. TEM images were obtained from a JEOL JEM-2010 transmission electron microscope with an accelerating voltage of 200 kV. UV-vis absorption spectra were taken on a Shimadzu Model UV-1700 spectrometer. Photoluminescence (PL) spectra were measured on a Perkin-Elmer LS 55 spectrofluorometer. All UV and PL spectra were collected at 24 ± 1 °C.

Synthesis of 4,4'-(2-(4-bromophenyl)-2-phenylethene-1,1-diyl)bis(methoxybenzene) (compound 1)

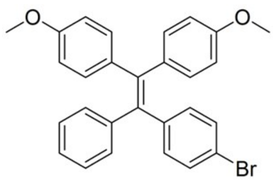
(1)

To a solution of bis(4-methoxyphenyl)methanone (3.8 g, 16 mmol), (4-bromophenyl)(phenyl)methanone (5.3 g, 20 mmol) and zinc dust (5.9 g, 91 mmol) in dry THF (80 mL), $TiCl_4$ (5.0 mL, 46 mmol) was added dropwise under argon atmosphere at 0 ^{o}C. After the addition was complete, the reaction was warmed up to room temperature and refluxed overnight. Then the reaction was quenched with saturated aqueous sodium bicarbonate (50 mL) and extracted with ethyl acetate (100 mL × 3). The combined extracts were washed with brine and dried over anhydrous magnesium sulphate. The solution was concentrated *in vacuo* and purified by column chromatography to give product **1** as a yellowish solid (3.0 g, 40% yield). ^{1}H NMR (300 MHz, $CDCl_3$) δ 7.16 – 7.10 (m, 2H), 7.08 –

6.97 (m, 4H), 6.97 – 6.88 (m, 3H), 6.88 – 6.76 (m, 6H), 6.62 – 6.49 (m, 4H), 3.68 (s, 3H), 3.65 (d, J = 1.1 Hz, 3H); ^{13}C NMR (151 MHz, $CDCl_3$) δ 158.42, 158.34, 143.93, 143.45, 140.93, 136.16, 136.08, 133.19, 132.69, 132.66, 131.49, 130.99, 127.95, 126.45, 120.16, 113.35, 113.17, 55.27, 55.24.

Synthesis of *N*^{2}, *N*^{5}-dimethoxy-*N*^{2},*N*^{5}-dimethylthiophene-2,5-dicarboxamide (compound 2)

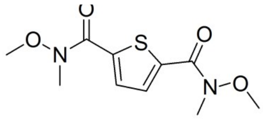

(2)

To a solution of thiophene-2,5-dicarboxylic acid (4.4 g, 20 mmol) and *N*,*O*-dimethylhydroxylamine hydrochloride (7.8 g, 80 mmol) in DMF (10 mL) was added HBTU (23 g, 60 mmol) and TEA (17 mL) at 0 ^{o}C. After 1 hr, the reaction was allowed to warm up to room temperature and stirred at room temperature overnight. After DMF removal by distillation *in vacuo*, the residue was extracted with ethyl acetate (100 mL × 3). The combined extracts were washed with brine (100 mL) and dried over anhydrous magnesium sulphate. The solution was concentrated *in vacuo*, and the residue was purified by column chromatography to give product **2** as a white solid (4.78 g, 92% yield). ^{1}H NMR (300 MHz, $CDCl_3$) δ 7.89 (s, 2H), 3.79 (s, 6H), 3.39 (s, 6H); ^{13}C NMR (75 MHz, $CDCl_3$) δ 161.85, 138.38, 133.18, 61.61, 33.03.

Synthesis of 5-(4-(2,2-bis(4-methoxyphenyl)-1-phenylvinyl)benzoyl)-*N*-methoxy-*N*-methylthiophene-2-carboxamide (compound 3)

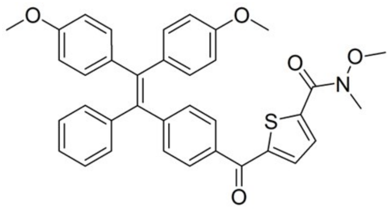

(3)

To a solution of compound **1** (1.7 g, 3.6 mmol) in dry THF (30 mL) was added butyllithium (1.6 M, 2.7 mL) at -78 ^{o}C. After the reaction was stirred at -78 ^{o}C for 1 hr, compound **2** (0.62 g, 2.4 mmol) in dry THF (9.0 mL) was added. Then the reaction was warmed to room temperature slowly and stirred at room temperature overnight. The reaction was quenched by aqueous ammonium chloride (50 mL) and extracted with ethyl acetate (60 mL × 3). The combined extracts were washed with brine and dried over anhydrous magnesium sulphate. After solvent removal, the residue was purified by column chromatography to give the product **3** as a yellow solid (0.66 g, 31% yield). ^{1}H NMR (400 MHz, $CDCl_3$) δ 7.93 (d, J = 4.1 Hz, 1H), 7.64 (d, J = 8.3 Hz, 2H), 7.54 (d, J = 4.0 Hz, 1H), 7.21 – 7.08 (m, 5H), 7.03 (dd, J = 7.6, 2.0 Hz, 2H), 6.98 – 6.89 (m, 4H), 6.72 – 6.57 (m, 4H), 3.82 (s, 3H), 3.74 (d, J = 3.5 Hz,

6H), 3.40 (s, 3H); ^{13}C NMR (101 MHz, $CDCl_3$) δ 188.37, 161.47, 158.64, 158.47, 149.63, 147.94, 143.70, 142.17, 138.78, 138.10, 135.85, 135.80, 134.93, 134.50, 132.91, 132.77, 132.69, 131.61, 131.49, 129.07, 128.03, 126.57, 113.35, 113.16, 61.96, 55.22, 55.20, 33.15. LRMS (ESI): m/z = 590.21 [M+H$^+$], calc for ($C_{36}H_{32}NO_5S$): m/z = 590.20; HRMS (ESI): m/z = 612.1813 [M+Na$^+$], calc for ($C_{36}H_{31}NNaO_5S$): m/z = 612.1815.

Synthesis of 5-(4-(2,2-bis(4-methoxyphenyl)-1-phenylvinyl)benzoyl)thiophene-2-carboxylic acid (compound 4)

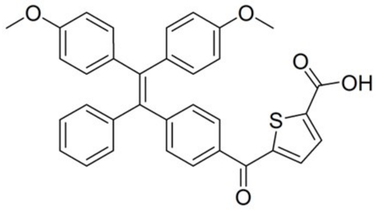

(4)

To a solution of compound **6** (95 mg, 0.16 mmol) in methanol-water (3:1, 50 mL) was added sodium hydroxide (32 mg, 0.81 mmol). Then the reaction was stirred at room temperature overnight. After methanol removal, the reaction mixture was acidified by 1 *N* HCl to pH 2~3. The mixture was extracted with dichloromethane (10 mL × 3). The combined organic layer was dried over anhydrous magnesium sulphate and concentrated *in vacuo* before column chromatography purification to give product **4** as a yellow oil (72 mg, 82% yield). ^{1}H NMR (300 MHz, $CDCl_3$) δ 8.30 (s, 1H), 7.85 (d, J = 4.0 Hz, 1H), 7.66 (d, J = 8.1 Hz, 2H), 7.59 (d, J = 4.0 Hz, 1H), 7.23 – 7.12 (m, 5H), 7.09 – 7.02 (m, 2H), 6.96 (d, J = 8.3 Hz, 4H), 6.69-6.64 (m, 4H), 3.75 (d, J = 3.6 Hz, 6H); ^{13}C NMR (75 MHz, $CDCl_3$) δ 187.33, 166.12, 158.48, 158.29, 149.85, 149.09, 143.47, 142.16, 137.83, 135.63, 135.56, 134.26, 134.19, 133.47, 132.61, 132.54, 131.54, 131.32, 128.97, 127.89, 126.44, 113.21, 113.00, 55.07, 55.03. LRMS (ESI): m/z = 545.03 [M-H$^+$], calc for ($C_{34}H_{25}O_5S$): m/z = 545.14; HRMS (ESI): m/z = 545.1434 [M-H$^+$], calc for ($C_{34}H_{25}O_5S$): m/z = 545.1428.

Synthesis of *N*-(3-azidopropyl)-5-(4-(2,2-bis(4-methoxyphenyl)-1-phenylvinyl)benzoyl)thiophene-2-carboxamide (compound 5)

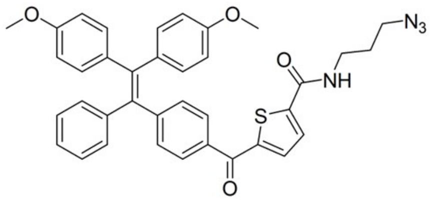

(5)

To a stirring solution of compound **4** (72 mg, 0.13 mmol) and HBTU (76 mg, 0.20 mmol) in DMF (3.0 mL) at room temperature was added 3-azidopropan-1-amine (26 mg, 0.26 mmol) and triethylamine (55 μL, 0. 40 mmol). The reaction was stirred at room temperature overnight. After the reaction was complete, DMF was removed *in*

vacuo. The residue was dissolved in dichloromethane (10 mL) and washed with water (5 mL) and brine (5 mL). The organic layer was dried over anhydrous magnesium sulphate and concentrated *in vacuo* before column chromatography purification to give the product **5** as a yellow oil (70 mg, 84% yield). ^{1}H NMR (400 MHz, $CDCl_3$) δ 7.63 (d, J = 8.1 Hz, 2H), 7.55 (s, 1H), 7.53 (s, 1H), 7.16 (m, 5H), 7.06 – 7.00 (m, 2H), 6.96-6.93 (m, 4H), 6.69 – 6.54 (m, 4H), 6.42-6.39 (m, 1H), 3.75 (s, 6H), 3.55 (q, J = 6.3 Hz, 2H), 3.46 (t, J = 6.3 Hz, 2H), 1.94-1.89 (m, 2H); ^{13}C NMR (151 MHz, $CDCl_3$) δ 187.48, 161.40, 158.69, 158.53, 149.83, 146.24, 144.68, 143.72, 142.28, 138.08, 135.86, 135.80, 134.70, 133.93, 132.81, 132.73, 131.70, 131.52, 129.07, 128.72, 128.08, 126.62, 113.41, 113.20, 55.29, 55.25, 49.74, 38.22, 28.79; LRMS (ESI): m/z = 628.9 [$M+H^+$], calc for ($C_{37}H_{32}N_4O_4S$): m/z = 629.2; HRMS (ESI): m/z = 651.2051 [$M+Na^+$], calc for ($C_{37}H_{32}N_4NaO_4S$): m/z = 651.2036.

Synthesis of *N*-(3-azidopropyl)-5-(1-(4-(2,2-bis(4-methoxyphenyl)-1-phenylvinyl)phenyl)-2,2-dicyanovinyl)thiophene-2-carboxamide (TPETP-N_3)

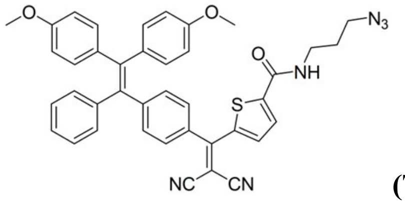

(TPETP-N_3)

To a solution of compound **5** (70 mg, 0.11 mmol) and malononitrile (9.6 mg, 0.14 mmol) in dry dichloromethane (2 mL) at 0 °C, pyridine (31 µL, 0.39 mmol) was added and stirred for 30 min at 0 °C. Then the mixture was kept at 40 °C for 4 hrs. The reaction was cooled to room temperature and quenched with water and extracted with dichloromethane (5 mL × 3). The combined extracts were dried over anhydrous magnesium sulphate. The solution was concentrated *in vacuo* to give residue which was purified by column chromatography to give product TPETP-N_3 as a red solid (65 mg, 88% yield). ^{1}H NMR (300 MHz, $CDCl_3$) δ 7.79 (d, J = 4.0 Hz, 1H), 7.52 (d, J = 4.2 Hz, 1H), 7.22 – 7.11 (m, 7H), 7.06 (dd, J = 7.5, 2.1 Hz, 2H), 7.00 – 6.89 (m, 4H), 6.77 – 6.59 (m, 4H), 6.40 (s, 1H), 3.75 (s, 6H), 3.55 (q, J = 6.4 Hz, 2H), 3.46 (t, J = 6.3 Hz, 2H), 1.99 – 1.82 (m, 2H); ^{13}C NMR (75 MHz, $CDCl_3$) δ 164.22, 160.50, 158.65, 158.45, 149.43, 146.85, 143.08, 142.71, 141.73, 137.60, 135.41, 135.05, 132.85, 132.72, 132.59, 131.71, 131.36, 129.25, 128.50, 128.04, 126.59, 114.11, 113.39, 113.30, 113.07, 55.21, 55.10, 49.58, 38.19, 28.56; LRMS (ESI): m/z = 699.2 [$M+Na^+$], calc for ($C_{40}H_{32}N_6NaO_3S$): m/z = 699.2; HRMS (ESI): m/z = 699.2163 [$M-H^+$], calc for ($C_{40}H_{32}N_6NaO_3S$): m/z = 699.2149.

Synthesis of TPETP-DEVD. TPETP-N_3 (5 mg, 7.38 µmol) and DEVD-alkyne (5.04 mg, 8.83 µmol) were firstly dissolved in DMSO/H_2O (v/v = 10:1) and mixed thoroughly. Into the mixture, sodium ascorbate (0.6 mg, 3.0

μmol) and copper sulphate (0.8 mg, 1.5 μmol) were added to initiate the click reaction and the mixture was shaken at room temperature for another 24 h. The product TPETP-DEVD was purified by prep-HPLC and further characterized by ^{1}H NMR and HRMS. ^{1}H NMR (400 MHz, DMSO-d_6), δ: 8.88 (t, J = 4.0 Hz, 1H), 8.52 (d, J = 8.0 Hz, 1H), 8.24 (d, J = 8.0 Hz, 1H), 7.96 (d, J = 8.0 Hz, 1H), 7.83 (d, J = 8.0 Hz, 1H), 7.80 (d, J = 4.0 Hz, 1H), 7.74 (s, 1H), 7.62 (d, J = 4.0 Hz, 1H), 7.30 (d, J = 8.0 Hz, 2H), 7.15-7.11 (m, 3H), 7.07-7.04 (m, 4H), 6.98 (d, J = 8.0 Hz, 2H), 6.85 (d, J = 8.0 Hz, 2H), 6.79 (d, J = 8.0 Hz, 2H), 6.66 (t, J = 8.0 Hz, 4H), 4.48 (t, J = 8.0 Hz, 1H), 4.36-4.27 (m, 4H), 4.09-4.05 (t, J = 8.0 Hz, 2H), 3.62 (d, J = 4.0 Hz, 6H, overlap with DMSO-d_6), 3.23-3.18 (m, 2H), 3.03-2.98 (dd, J = 4.0 Hz, 1H), 2.88-2.82 (m, 1H), 2.78-2.72 (m, 1H), 2.66-2.55 (m, 2H), 2.5 (m, 2H, overlap with DMSO-d_6), 2.26-2.13 (m, 2H), 2.03-1.96 (m, 2H), 1.89-1.82 (m, 2H), 1.71-1.65 (m, 1H), 0.76-0.70 (m, 6H). HRMS (ESI-MS): m/2z $[M-2H]^{2-}$ calcd for $C_{63}H_{67}N_{12}O_{14}S$: 622.2198; found: 622.2204.

Synthesis of Cou-DEVD-TPETP. TPETP-DEVD (4 mg, 2.68 μmol) was firstly dissolved in 500 μL dry DMSO and mixed with DIPEA (1 μL), then Cou-NHS (1.2 mg, 3.22 μmol) was added into the mixture and the reaction was continued for another 24 h. The product was purified by prep-HPLC to yield Cou-DEVD-TPETP as an orange powder (2.6 mg, 52% yield), which was further characterized by ^{1}H NMR and HRMS. ^{1}H NMR (400 MHz, DMSO-d_6), δ (TMS, ppm): 9.11 (d, J = 8.0 Hz, 1H), 8.85 (m, 1H), 8.57 (d, J = 4.0 Hz, 1H), 8.19-8.12 (dd, J = 8.0 Hz, 2H), 7.95 (d, J = 8.0 Hz, 1H), 7.78 (t, J = 8.0 Hz, 2H), 7.61 (m, J = 12.0 Hz, 3H), 7.29 (d, J = 8.0 Hz, 2H), 7.13-7.03 (m, 7H), 6.97-6.94 (d, J = 12.0 Hz, 2H), 6.84-6.72 (m, 5H), 6.65-6.56 (m, 5H), 4.72 (d, J = 4.0 Hz, 1H), 4.44 (d, J = 8.0 Hz, 1H), 4.29 (m, 4H), 4.02 (t, J = 8.0 Hz, 1H), 3.62-3.59 (m, 6H, overlap with DMSO-d_6), 3.2 (m, 3H), 3.04-2.99 (m, 1H), 2.88-2.80 (m, 1H), 2.65-2.60 (m, 3H), 2.48 (m, 4H, overlap with DMSO-d_6), 2.19-2.13 (m, 2H), 2.00-1.97 (m, 2H), 1.89-1.82 (m, 2H), 1.74-1.63 (m, 1H), 1.07-1.05 (t, J = 8.0 Hz, 6H), 0.71 (t, J = 8.0 Hz, 6H). HRMS (ESI-MS): m/z $[M+H]^+$ calcd for $C_{77}H_{80}N_{13}O_{17}S$: 1490.5510; found: 1490.5530.

General procedure for enzymatic assay. DMSO stock solutions of Cou-DEVD-TPETP were diluted with a mixture of DMSO and PBS (v/v = 1/99) to 10 μM. Next, the probe was incubated with caspase-3 at 37 °C and the change of fluorescence intensity of Cou and TPETP were measured. The PL spectra of Cou were collected from 425 to 550 nm under excitation at 405 nm; the PL spectra of TPETP were collected from 600 to 780 nm under excitation at 405 nm.

Kinetic assay of Cou-DEVD-TPETP. The assay was studied following a method reported earlier.[1] Briefly, appropriate dilutions of Cou-DEVD-TPETP were added to reaction mixtures containing 100 pM of enzyme and buffer in a total volume of 50 μL. Liberation of TPETP residue was monitored continuously at 37 °C using a

BioTek Synergy 4 plate reader. Kinetic constants were computed by direct fitting the data to the Michaelis-Menton Equation using a non-linear regression via GraphPad Prism software. The value was taken in mean ± S.D.

Cell Culture. Human cervix carcinoma HeLa cells and human breast cancer MDA-MB-231 cells were provided by American Type Culture Collection (ATCC). The cells were cultured in DMEM medium containing 100 μg mL^{-1} streptomycin, 10% heat-inactivated FBS, 100 U mL^{-1} penicillin, and maintained in a humidified incubator with 5% CO_2 at 37 °C.

Confocal Imaging. HeLa and MDA-MB-231 cells were cultured in 8-well chambers (Thermo Scientific) at 37 °C. After 80% confluence, the culture medium was removed and washed twice with 1× PBS. After incubation with the probe for 4 h at 37 °C, the cells were washed twice with 1× PBS buffer and treated with 1 μM apoptosis inducers (STS, cisplatin or DOX) for different time. The cells were then washed twice with 1× PBS and the cell nuclei were live stained with SYTO® orange, following the standard protocols of the manufacturer (Life Technologies) and imaged immediately by confocal microscope (CLSM, Zeiss LSM 410, Jena, Germany). For colocalization with active caspase-3 antibody, the cells were fixed with 3.7% formaldehyde in 1× PBS for 15 min at room temperature, washed twice with cold 1× PBS again, and permeabilized with 0.1% Triton X-100 in 1× PBS for 10 min. The cells were then blocked with 2% BSA in 1× PBS for 30 min and washed twice with 1× PBS. The cells were subsequently incubated with a mixture of anti-caspase-3 antibody/PBS (v/v = 1/99) for 1 h at room temperature, washed once with 1× PBS buffer, and then incubated with mouse anti-rabbit IgG-TR (0.8 μg mL^{-1}) in 1× PBS for 1 h, followed by washing with 1× PBS. For SYTO® orange imaging, the excitation wavelength was 543 nm and the emission filter was 610−640 nm; for Cou-DEVD detection, the excitation wavelength was 405 nm, and the emission filter was 505−525 nm; for TPETP residue detection, the excitation was 405 nm and the emission was collected above 650 nm.

Flow Cytometry Study. HeLa and MDA-MB-231 cells in 24-well plate (Costar, IL, USA) were precultured overnight and incubated with the probe for the designated time and treated with 1 μM apoptosis inducers (STS, cisplatin or DOX) for different time. After incubation, the cells were washed with 1× PBS and treated with trypsin, washed with medium twice and subjected to flow cytometry analysis using Cyan-LX (DakoCytomation). The cells without any treatment were used as control. The mean fluorescence was determined by counting 10,000 events.

Cytotoxicity Studies. MTT assays were used to assess the cell viability of HeLa and MDA-MB-231 cells after incubation with the probe. The cells in 96-well plates (Costar, IL, USA) were incubated with the probe for

designated time in the dark. The cells were further incubated in fresh medium for 48 h and washed with 1× PBS. Then MTT in 1× PBS solution (100 μL, 0.5 mg mL^{-1}) was added into each well. After incubation for 3 h, the supernatant was discarded and the precipitate was dissolved in DMSO (100 μL) with gentle shaking. The absorbance of MTT at 570 nm was monitored by the microplate reader (Genios Tecan). The cells without any treatment were used as control.

1. (a) R. A. Copeland, *Enzymes: A Practical Introduction to Structure, Mechanism, and Data Analysis*, WILEY-VCH, New York, 2000; (b) M. Hu, L. Li, H. Wu, Y. Su, P.-Y. Yang, M. Uttamchandani, Q.-H. Xu, S. Q. Yao, *J. Am. Chem. Soc.* 2011, **133**, 12009-12020.

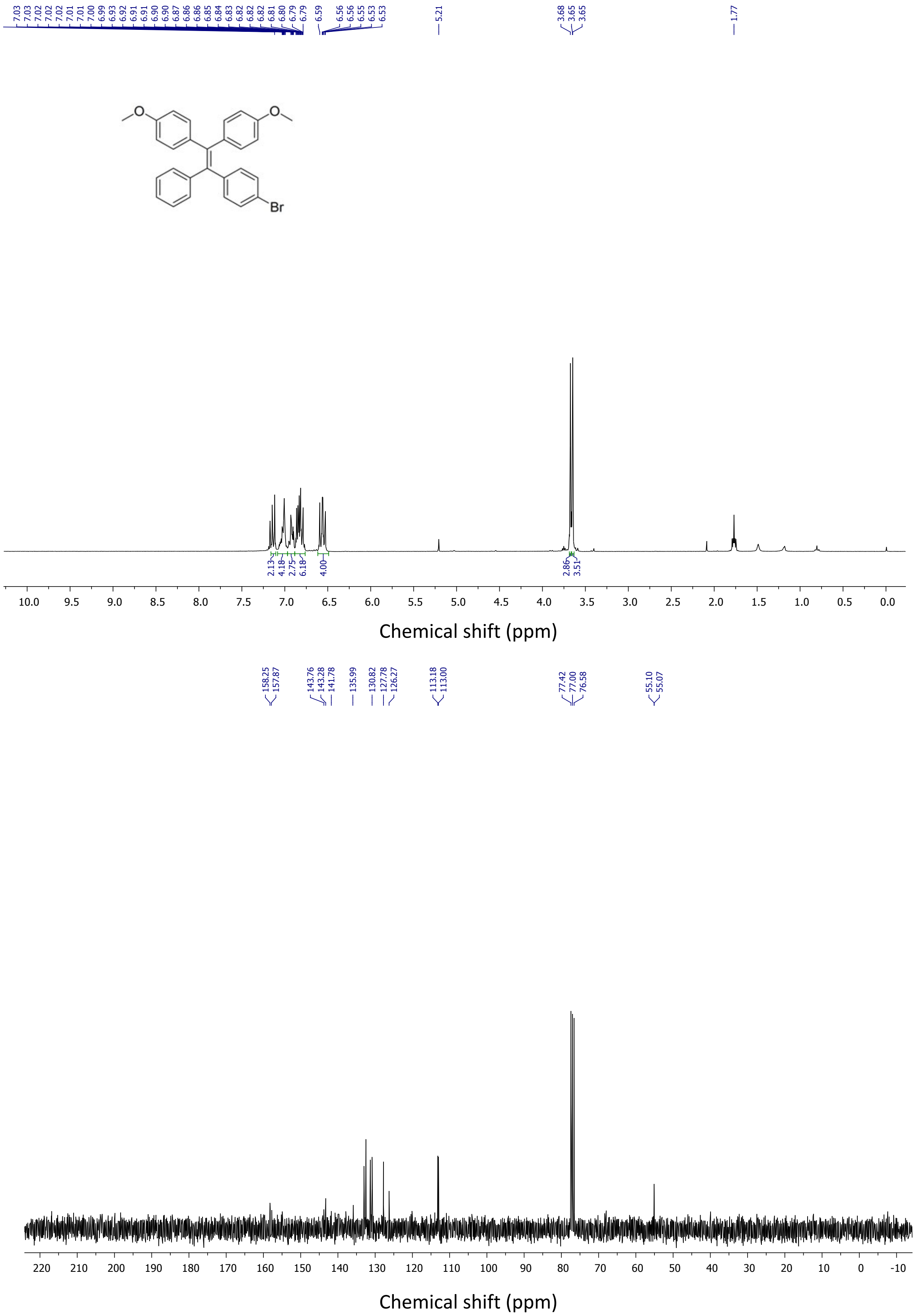

Figure S1. ^{1}H NMR and ^{13}C NMR spectra of compound **1** in $CDCl_3$.

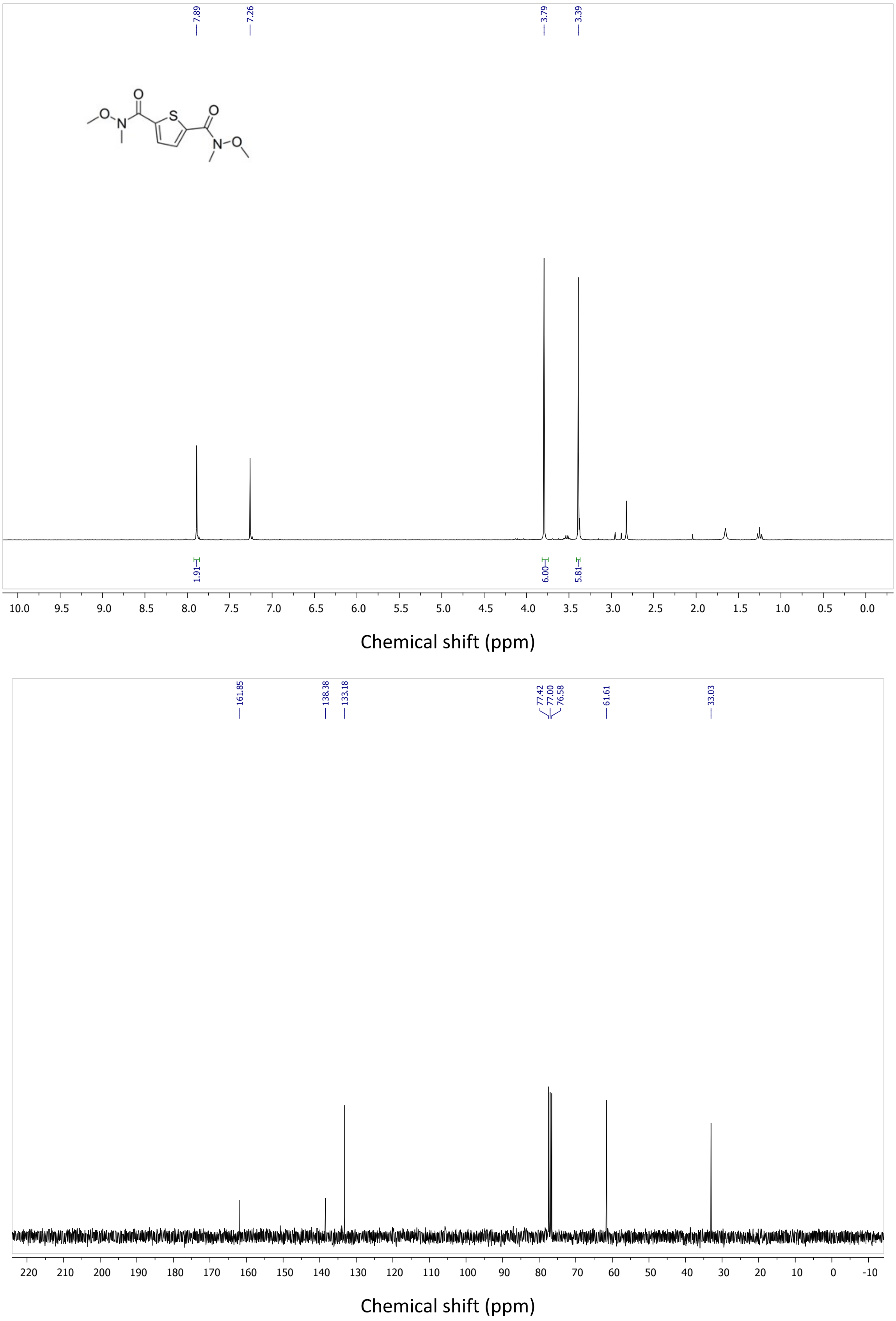

Figure S2. ^{1}H NMR and ^{13}C NMR spectra of compound **2** in $CDCl_3$.

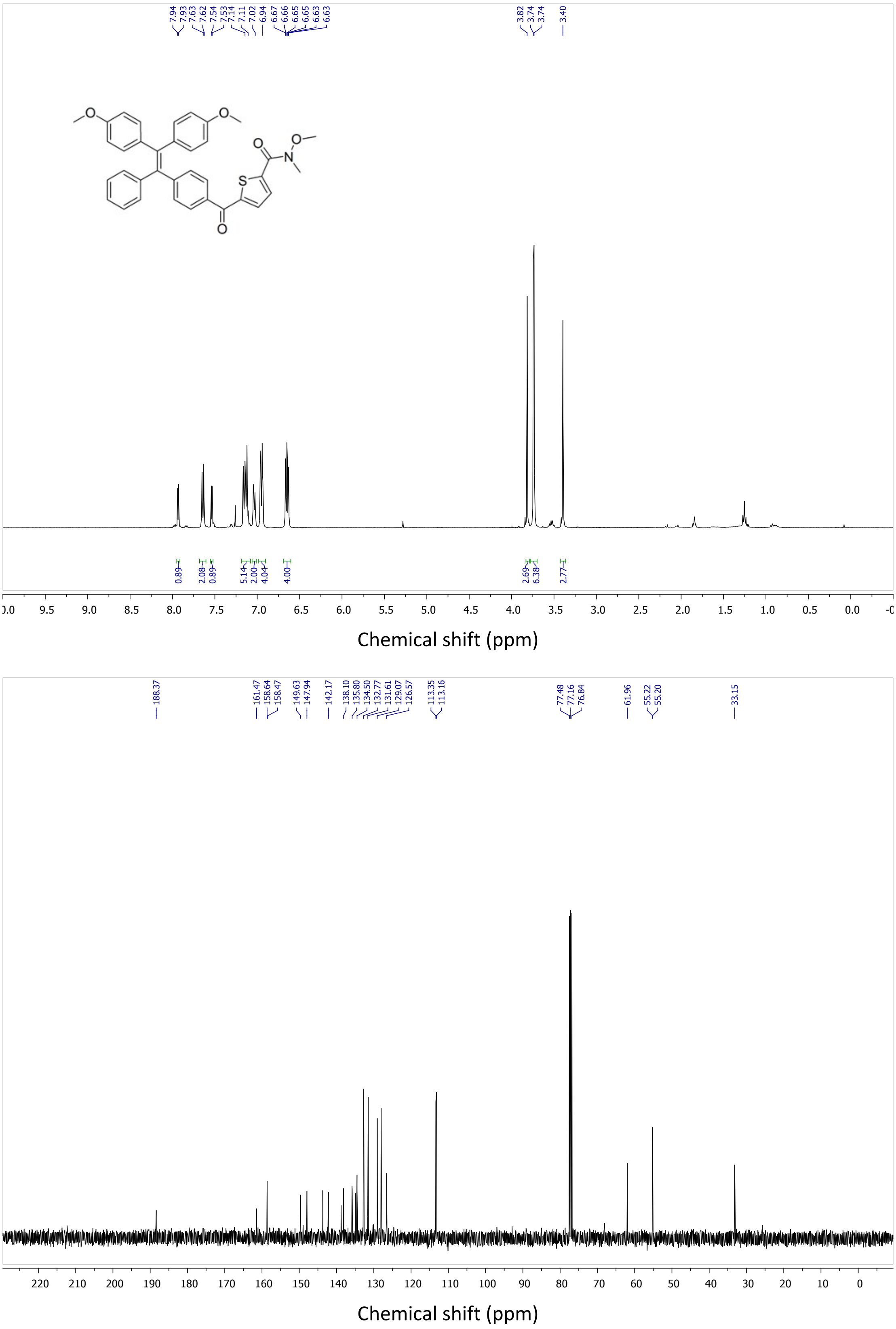

Figure S3. ^{1}H NMR and ^{13}C NMR spectra of compound **3** in $CDCl_3$.

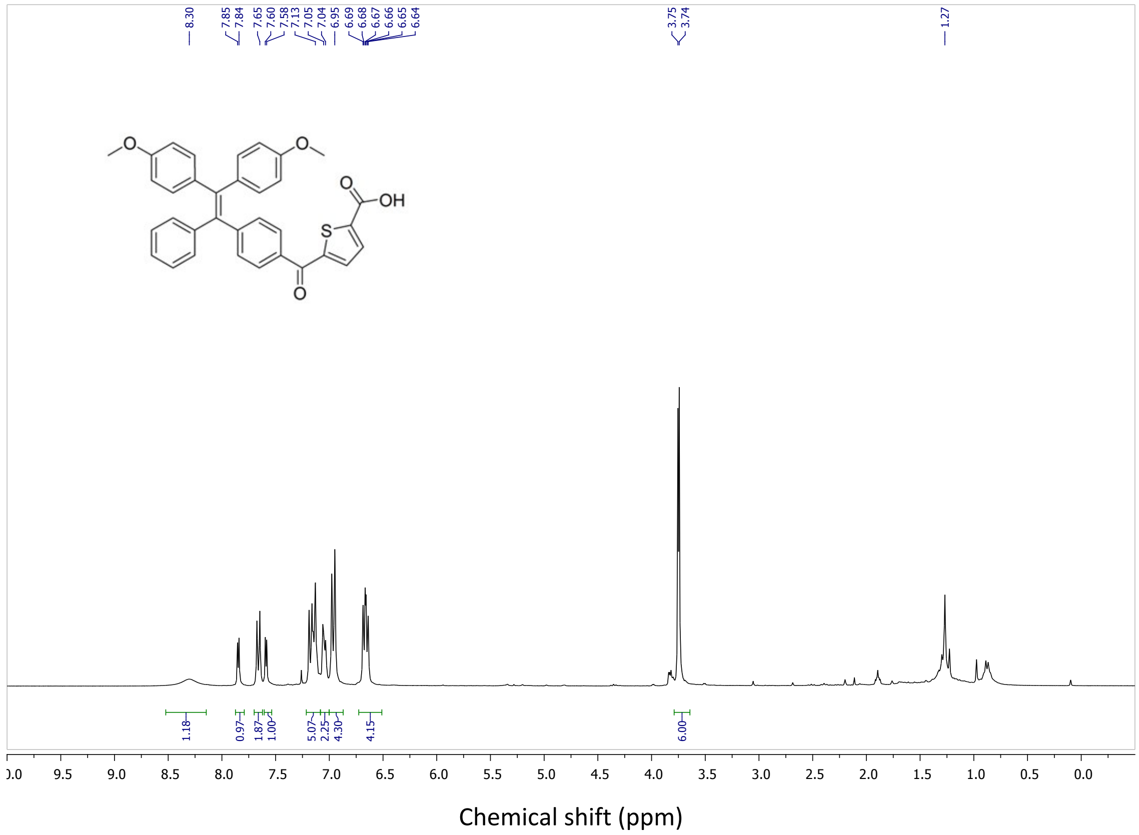

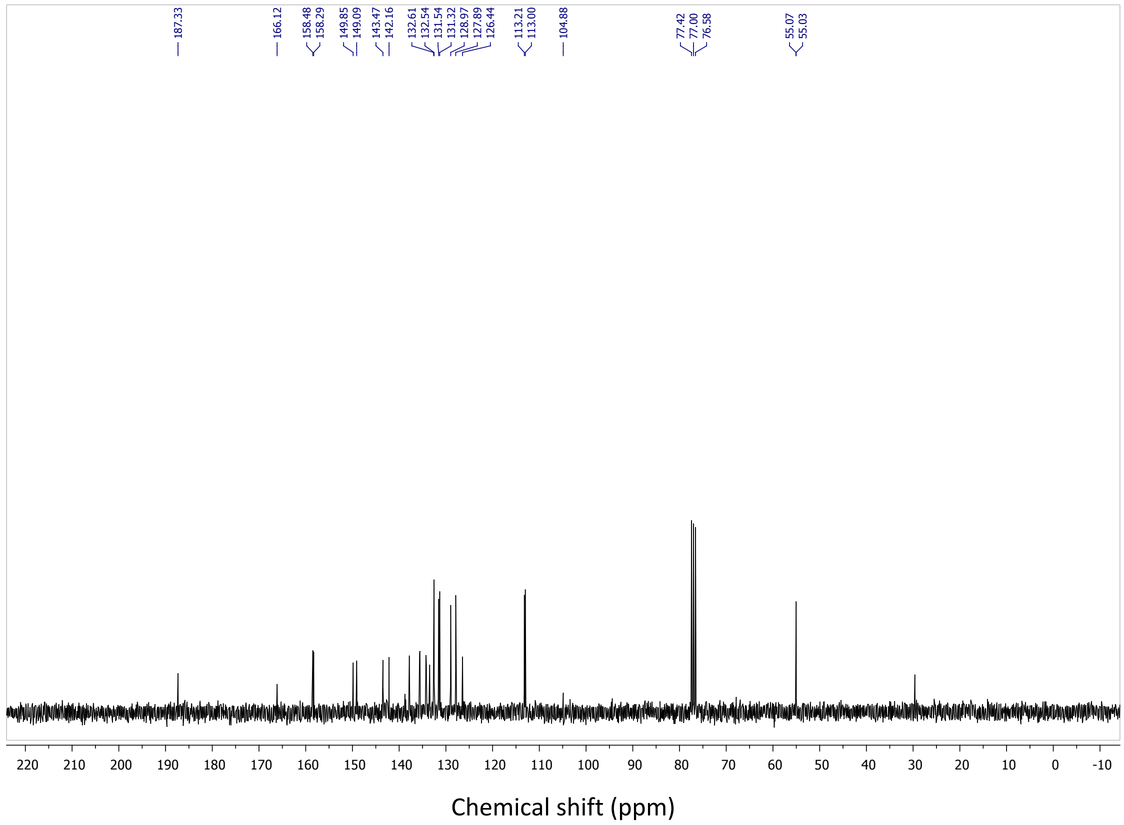

Figure S4. ^{1}H NMR and ^{13}C NMR spectra of compound **4** in $CDCl_3$.

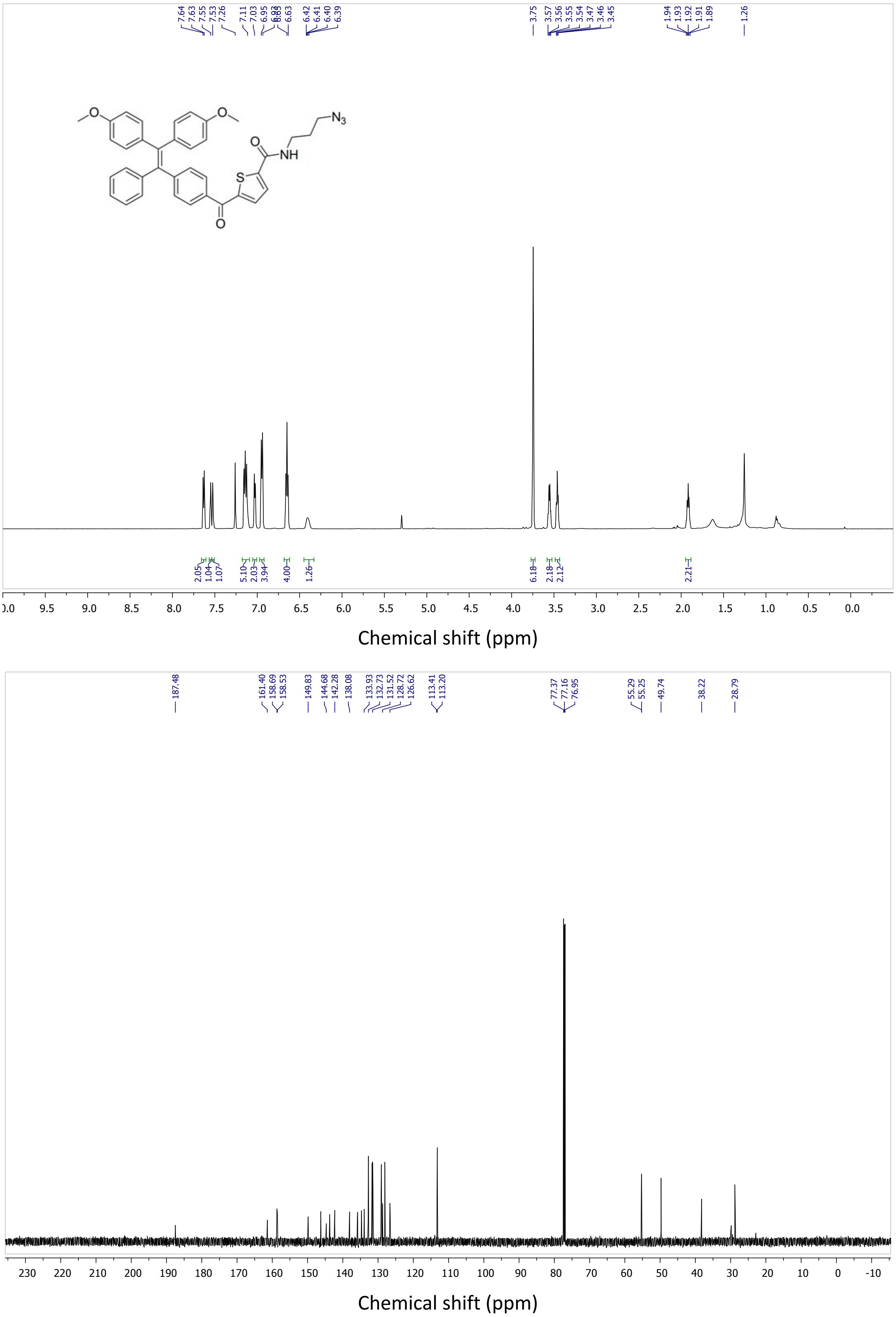

Figure S5. ^{1}H NMR and ^{13}C NMR spectra of compound **5** in $CDCl_3$.

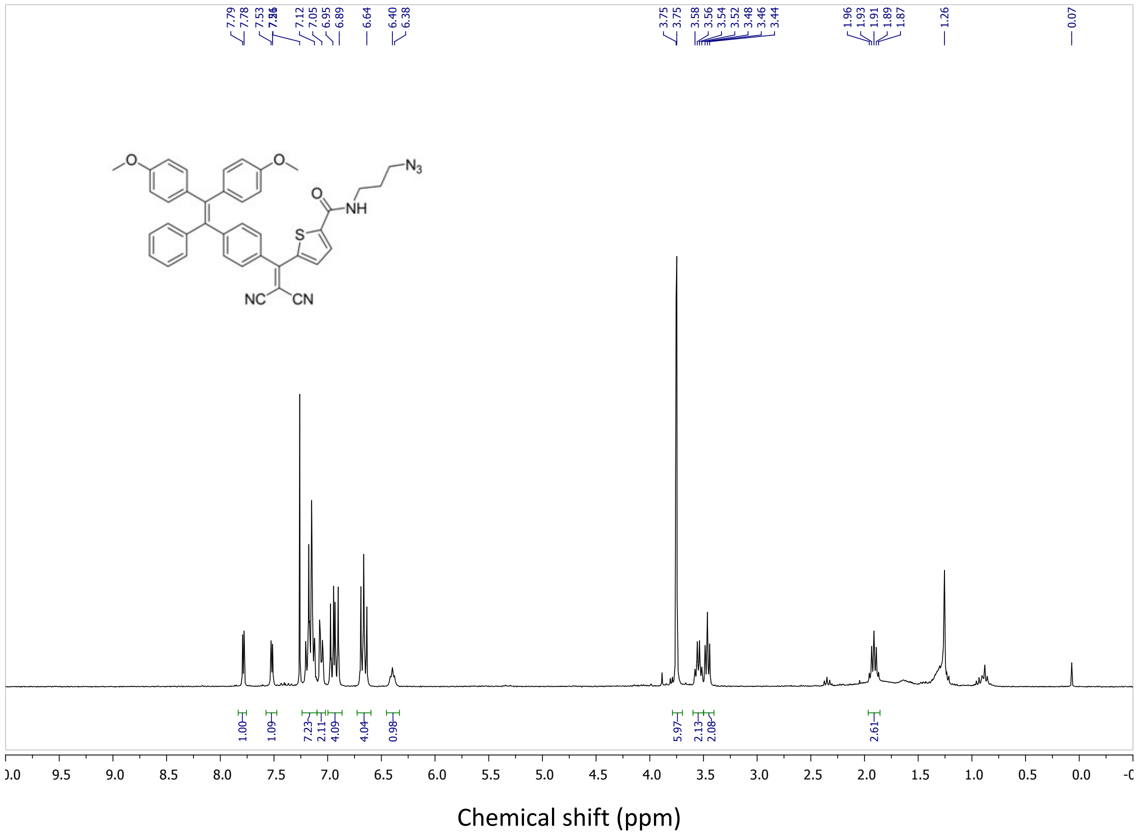

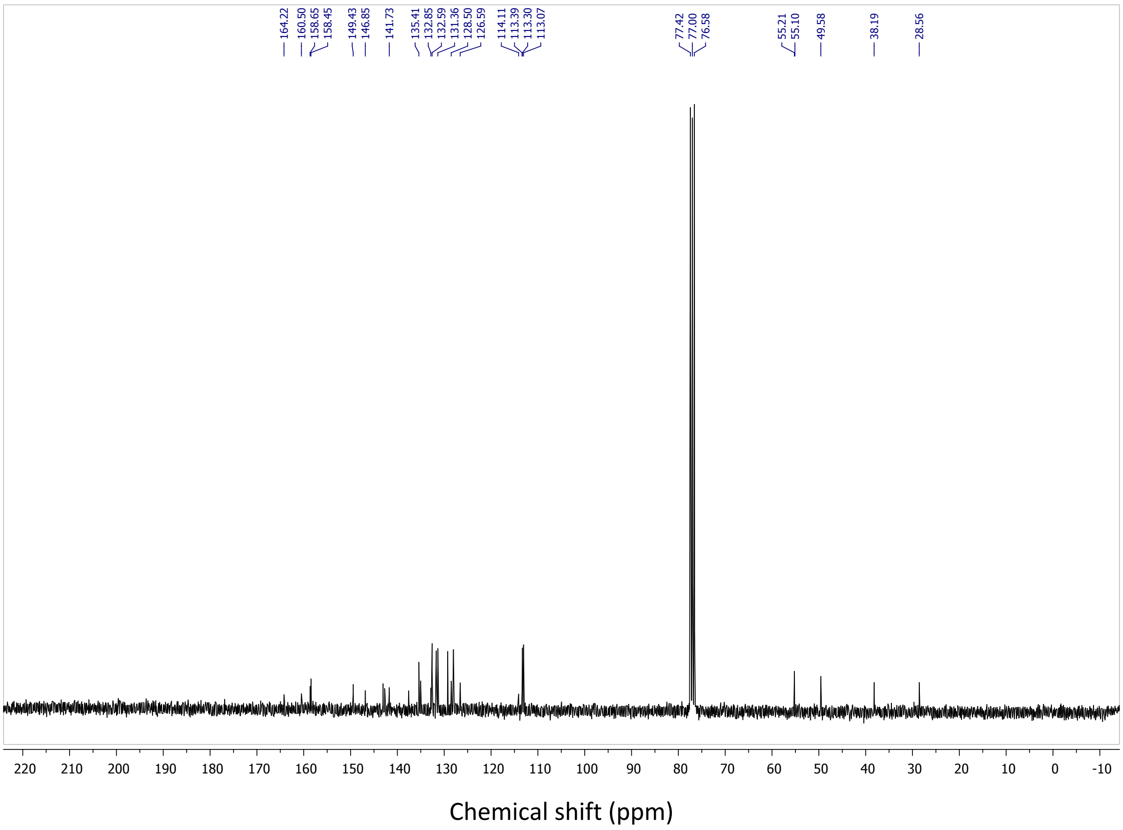

Figure S6. ^{1}H NMR and ^{13}C NMR spectra of TPETP-N_3 in $CDCl_3$.

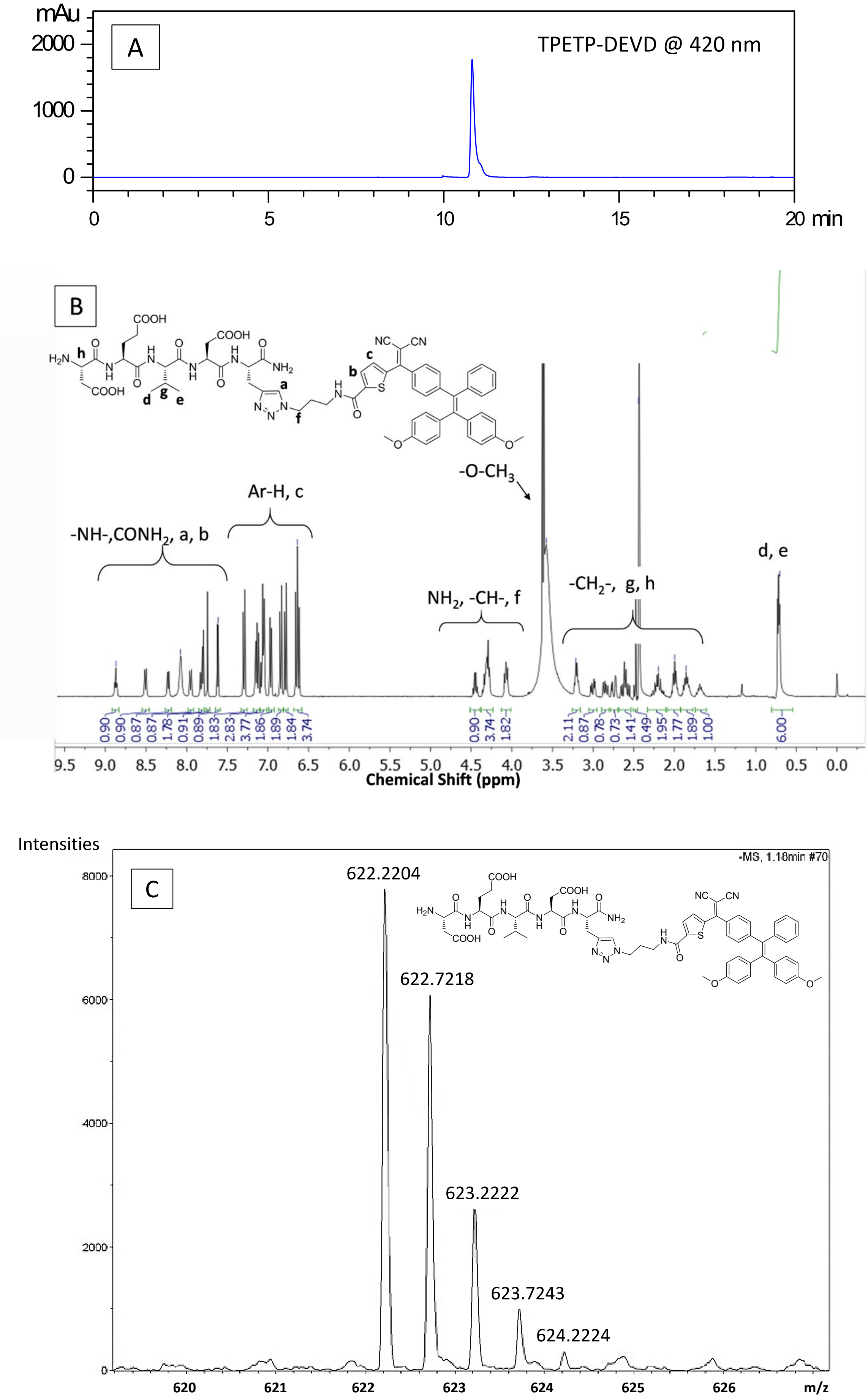
A
TPETP-DEVD @ 420 nm
mAu
2000
1000
0
0
5
10
15
20 min
B
-O-CH3
Ar-H, c
-NH-,CONH2, a, b
NH2, -CH-, f
-CH2-, g, h
d, e
Chemical Shift (ppm)
C
Intensities
-MS, 1.18min #70
622.2204
622.7218
623.2222
623.7243
624.2224
m/z

Figure S7. (A) HPLC spectrum of TPETP-DEVD; (B) ^{1}H NMR spectrum of TPETP-DEVD in DMSO-d_6; (C) High resolution mass spectrum (ESI-MS) of TPETP-DEVD. ($C_{63}H_{64}N_{12}O_{14}S$, $[M-2H]^{2-}$,Calcd: 622.2198, measured: 622.2204).

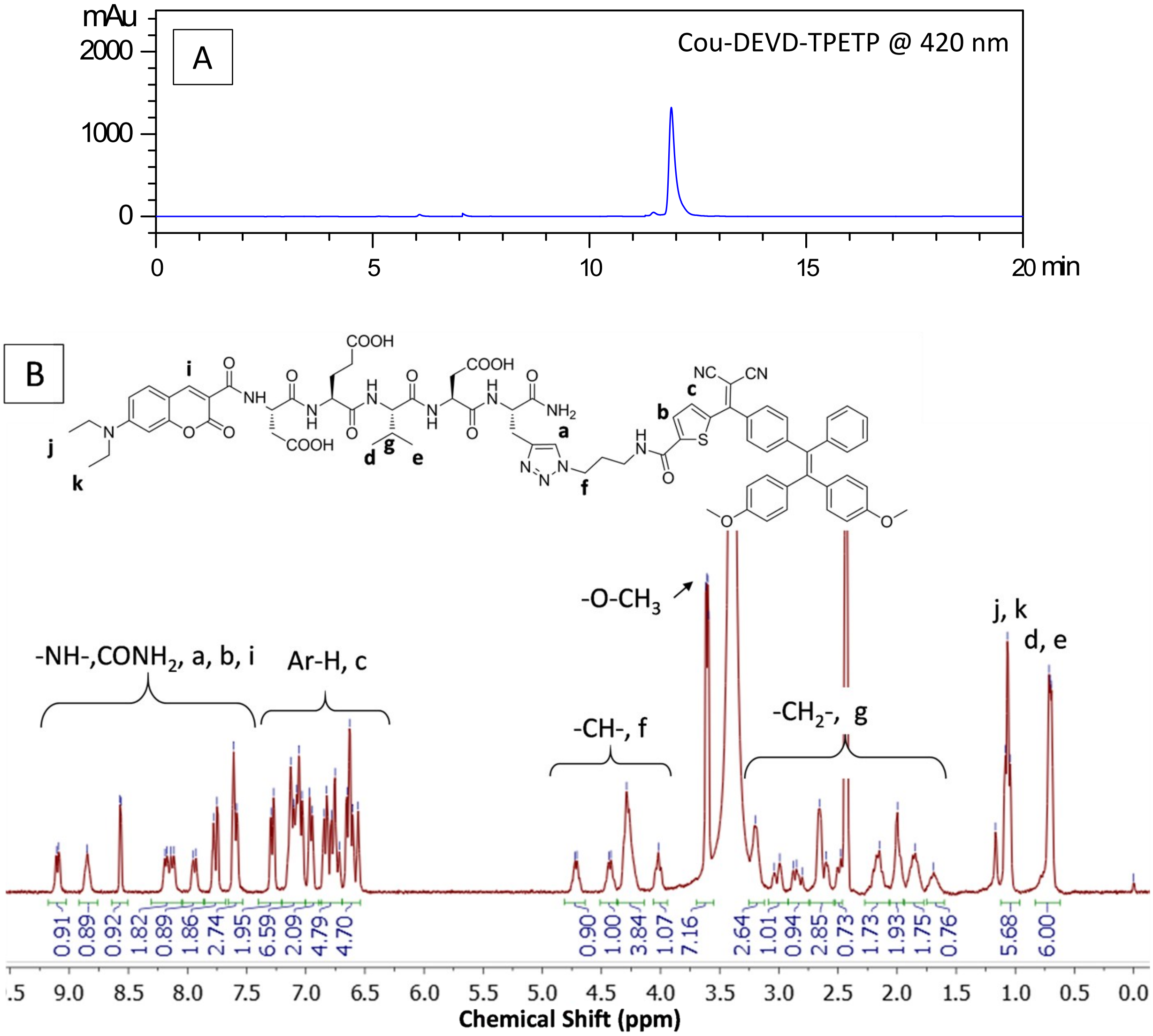

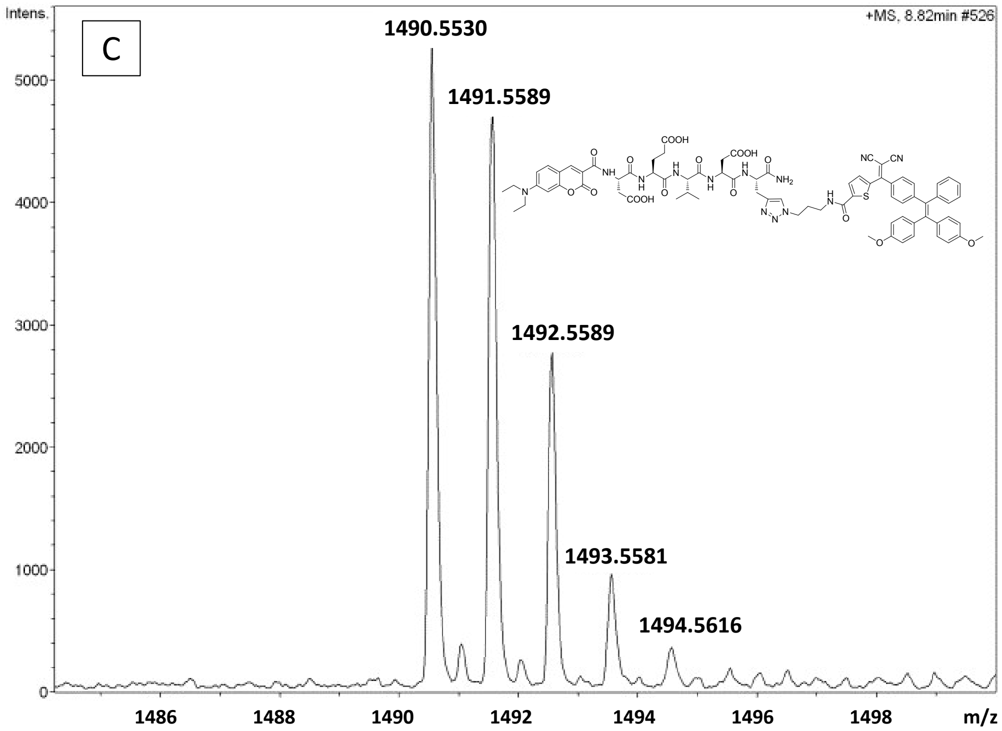

Figure S8. (A) HPLC spectrum of Cou-DEVD-TPETP; (B) ^{1}H NMR spectrum of Cou-DEVD-TPETP in DMSO-d_6; (C) High resolution mass spectrum (ESI-MS) of Cou-DEVD-TPETP, $C_{77}H_{80}N_{13}O_{17}S$, $[M+H]^+$ calcd: 1490.5510, Measured: 1490.5530.

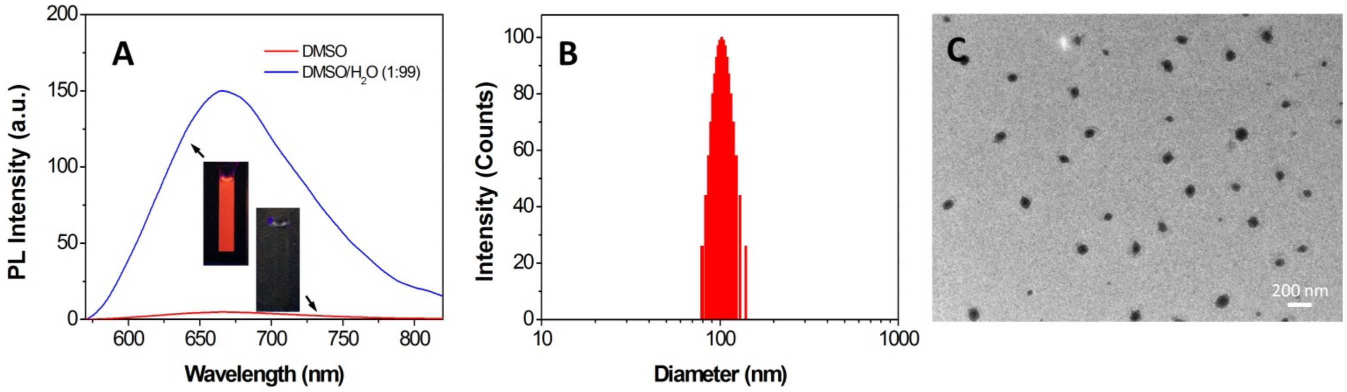

Figure S9. (A) Photoluminescence (PL) spectrum of TPETP-N_3 (10 μM) in DMSO or DMSO/water mixtures (v/v = 1/99). Inset: the digital photographs of TPETP-N_3 (10 μM) in DMSO or DMSO/water mixtures (v/v = 1/99) under illumination of a UV lamp at 365 nm. (B) Laser light scattering data and (C) TEM image of TPETP-N_3 (10 μM) in DMSO/water mixtures (v/v = 1/99).

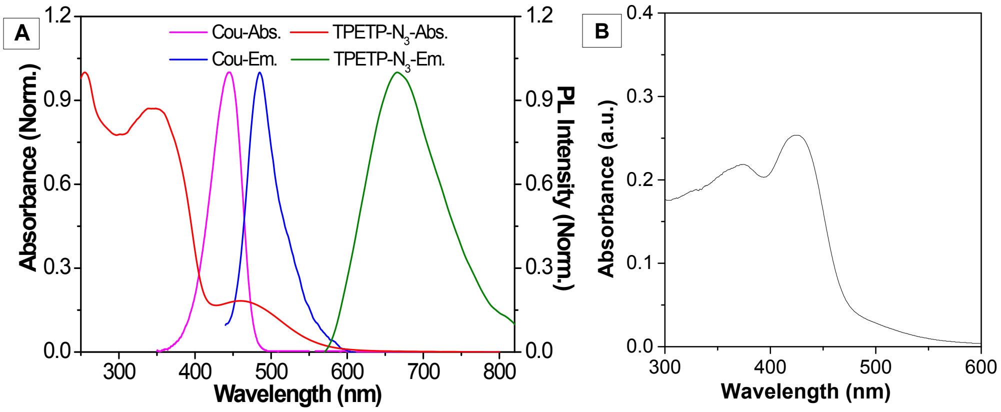

Figure S10. (A) Normalized UV-vis absorption and PL spectra of Cou and TPETP-N_3 in DMSO/PBS buffer (v/v = 1/99); (B) UV-vis absorption spectra of the probe Cou-DEVD-TPETP.

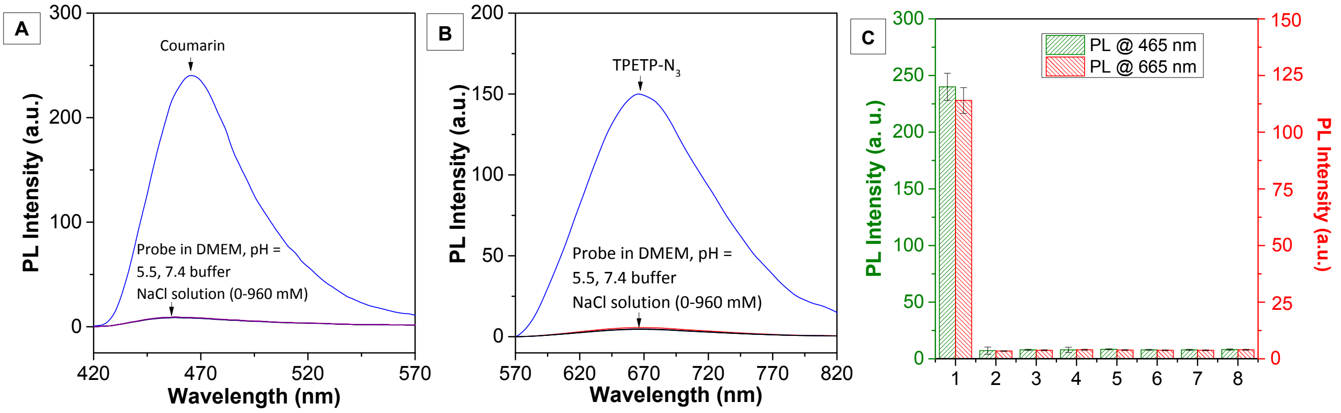

Figure S11. PL spectra of the Cou-DEVD-TPETP (10 μM) monitored at 420-570 nm and 570-820 nm upon excitation at 405 nm referenced against free coumarin and TPETP-N_3 in water containing [NaCl] ranging from 0, 250, 500, 960 mM, in cell culture medium (DMEM) or in buffer solutions with pH of 7.4 and 5.5 (C, DMSO/PBS buffer and DMSO/acetate buffer, v/v = 1/99), respectively (Entry 1-8).

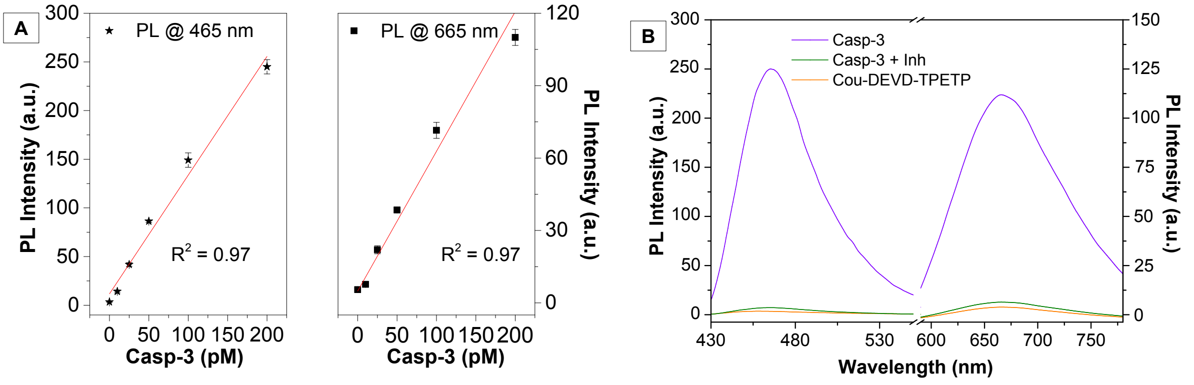

Figure S12. (A) PL intensities monitored at 465 nm and 665 nm for Cou-DEVD-TPETP (10 μM) probe upon incubation with caspase-3 at different concentrations; (B) PL spectra of Cou-DEVD-TPETP without and with treatment of caspase-3 in the presence and absence of inhibitor 5-[(S)-(+)-2-(methoxymethyl)pyrrolidino]

sulfonylisatin (10 μM). (λ_{ex}: 405 nm, emission collected from 430 – 550 nm is from the Cou-DEVD and that at 590 – 780 nm is from the TPETP residue).

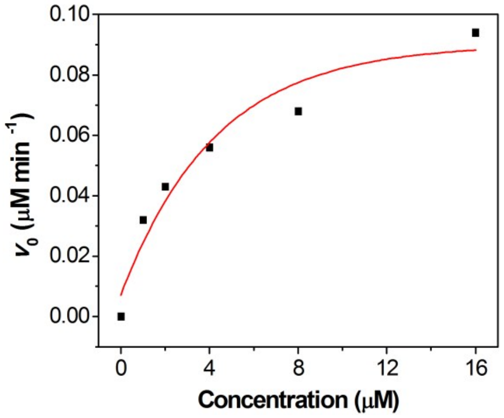

Figure S13. Michaelis-Menton plot for the hydrolysis of Cou-DEVD-TPETP at different concentrations by caspase-3 (100 pM).

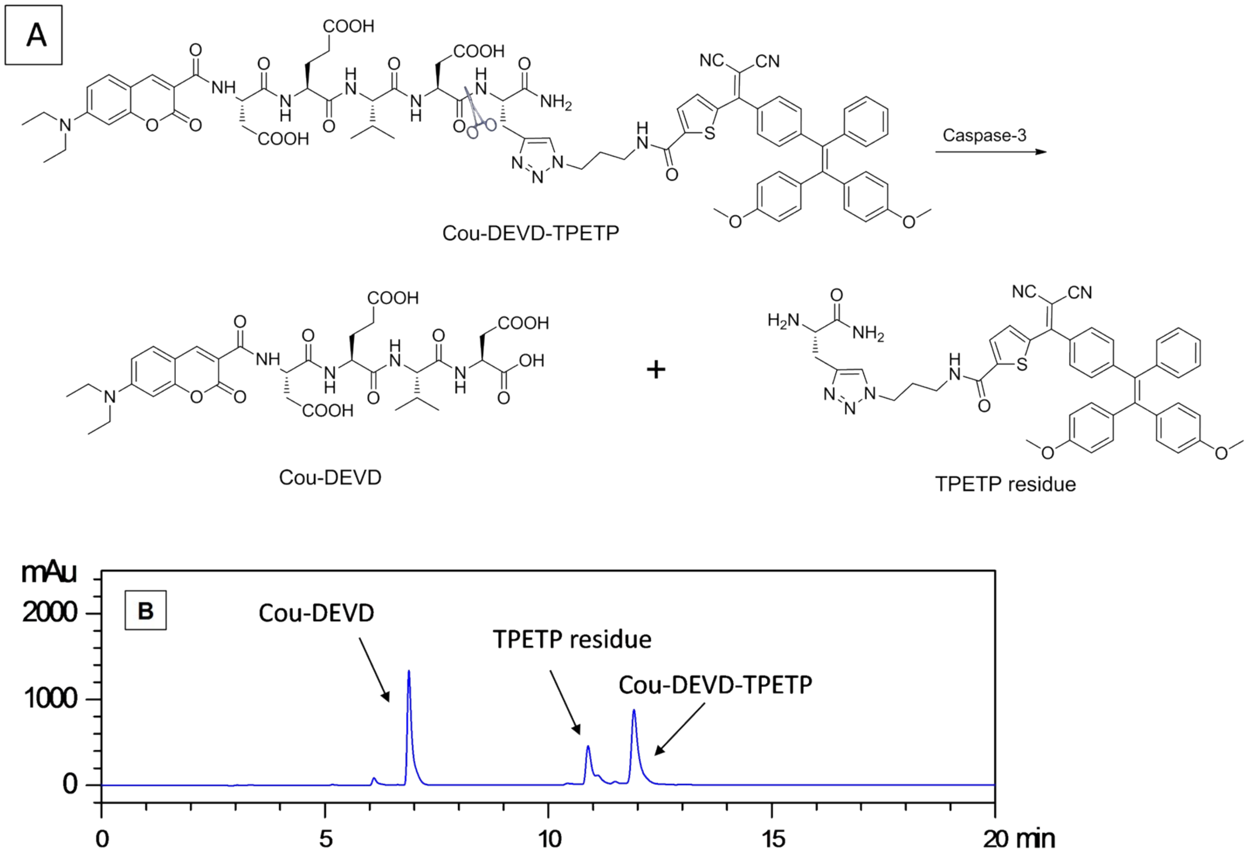

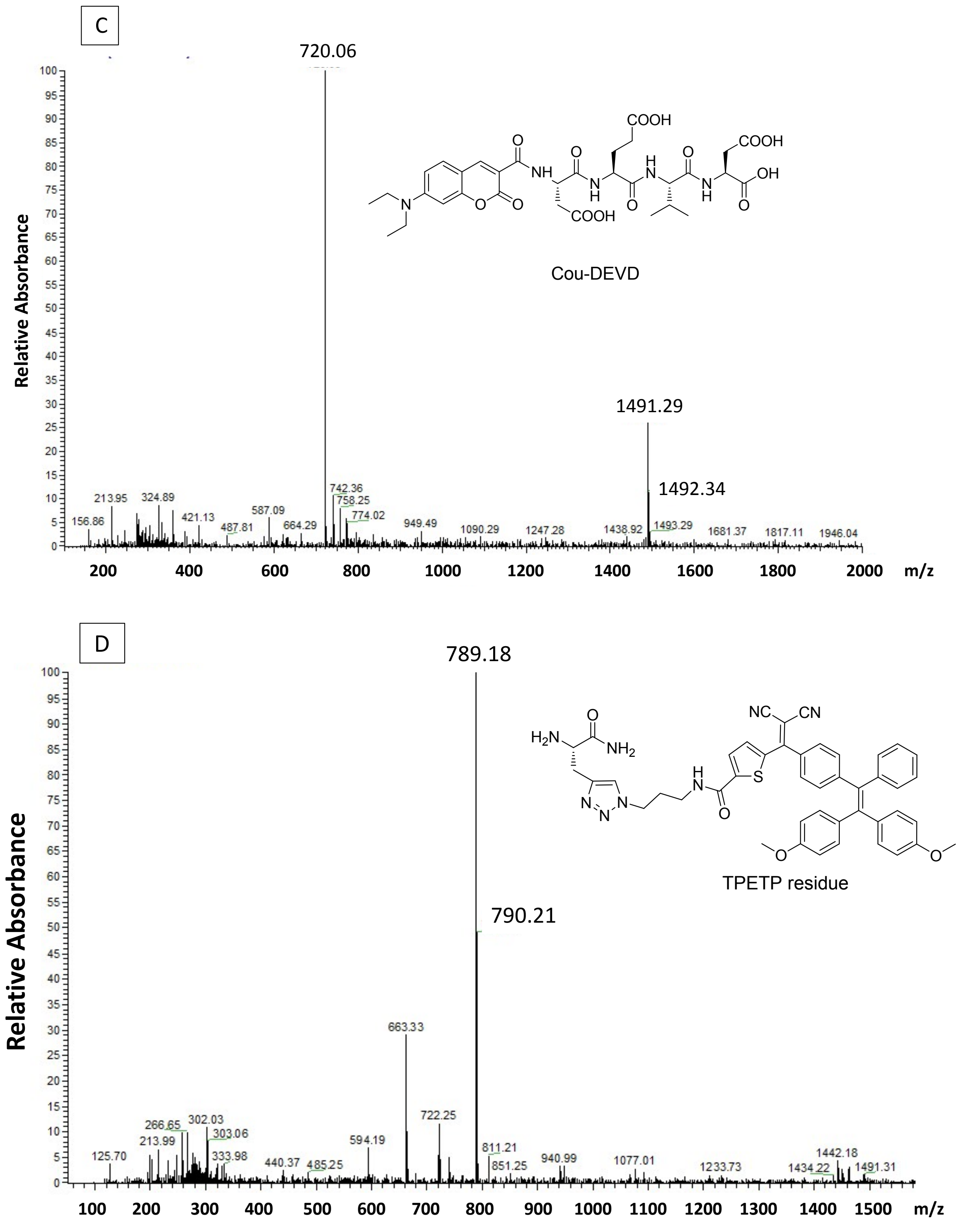

Figure S14. The caspase-catalyzed hydrolysis of Cou-DEVD-TPETP (t = 1 h) monitored by reverse phase HPLC; Mass spectra (ESI-MS) of Cou-DEVD ($[M+H]^+$ calcd: 720.28, Measured: 720.06) and TPETP residue ($[M+H]^+$ calcd: 788.29, Measured: 789.18).

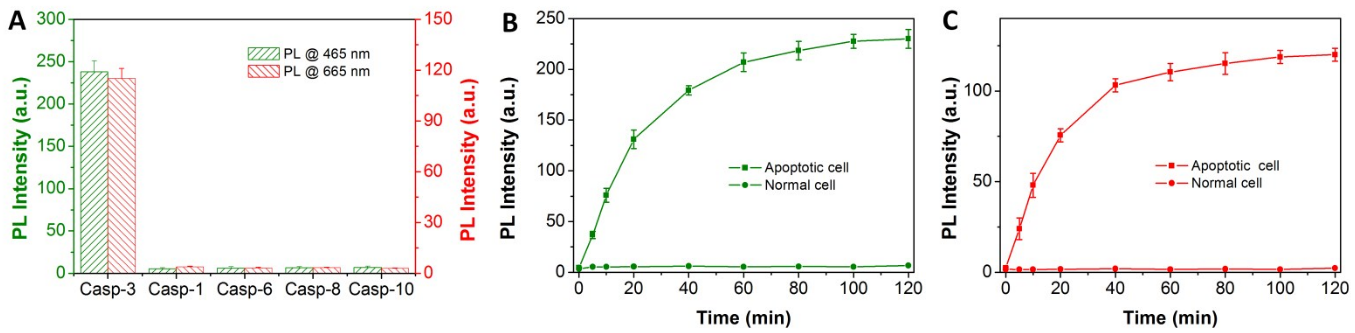

Figure S15. (A) PL intensities monitored at 465 nm and 665 nm for Cou-DEVD-TPETP in responsive to different caspase enzymes. (B, C) Time-dependent PL intensity changes of Cou-DEVD-TPETP in apoptotic and normal HeLa cell lysate (B) Monitored at 465 nm, and (C) monitored at 665 nm. Data represent mean values ± standard deviation, n = 3.

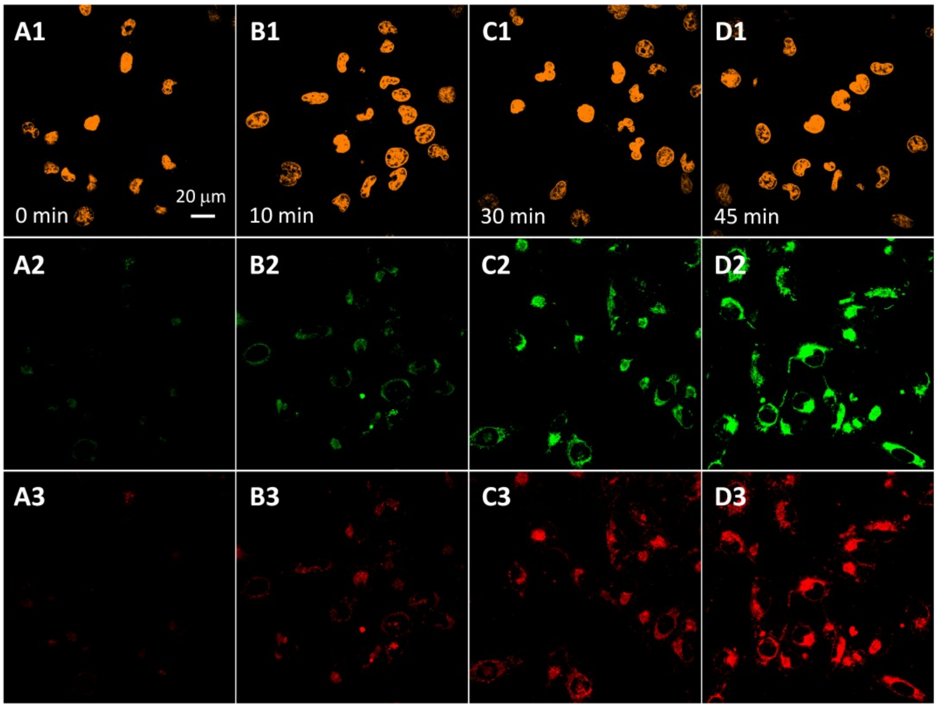

Figure S16. Confocal images of Cou-DEVD-TPETP (10 µM) incubated MDA-MB-231 cells treated with STS (1 µM) for (A) 0 min, (B) 10 min, (C) 30 min and (D) 45 min. Orange fluorescence (nucleus dyed with SYTO® orange, A1-D1, E_x: 543 nm, E_m: 610-640 nm); green fluorescence (A2-D2, E_x: 405 nm; E_m: 505-525 nm); red fluorescence (A3-D3, E_x: 405 nm, E_m: > 650 nm).

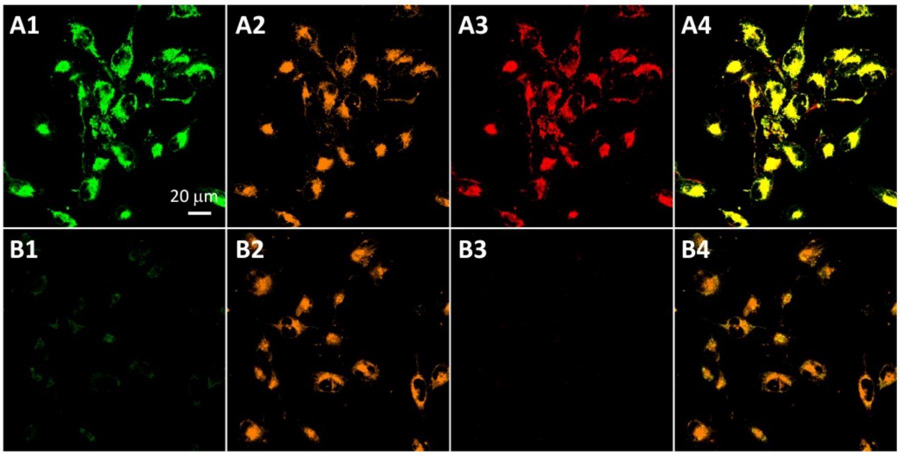

Figure S17. Confocal images of apoptotic MDA-MB-231 cells treated with Cou-DEVD-TPETP (10 μM) in the absence (A) and (B) presence of inhibitor and stained with anti-caspase-3 primary antibody and a Texas Red-labeled secondary antibody. Green fluorescence (Cou-DEVD, A1, B1, E_x: 405 nm; E_m: 505-525 nm); orange fluorescence (Texas Red, A2, B2, E_x: 543 nm, E_m: 610−640 nm); red fluorescence (TPETP residue, A3, B3, E_x: 405 nm, E_m: > 650 nm); A4, B4 are the overlay of images A1-A3 and B1-B3, respectively. Due to the low absorbance of TPETP residue at 543 nm, its spectral overlap with Texas Red is negligible.

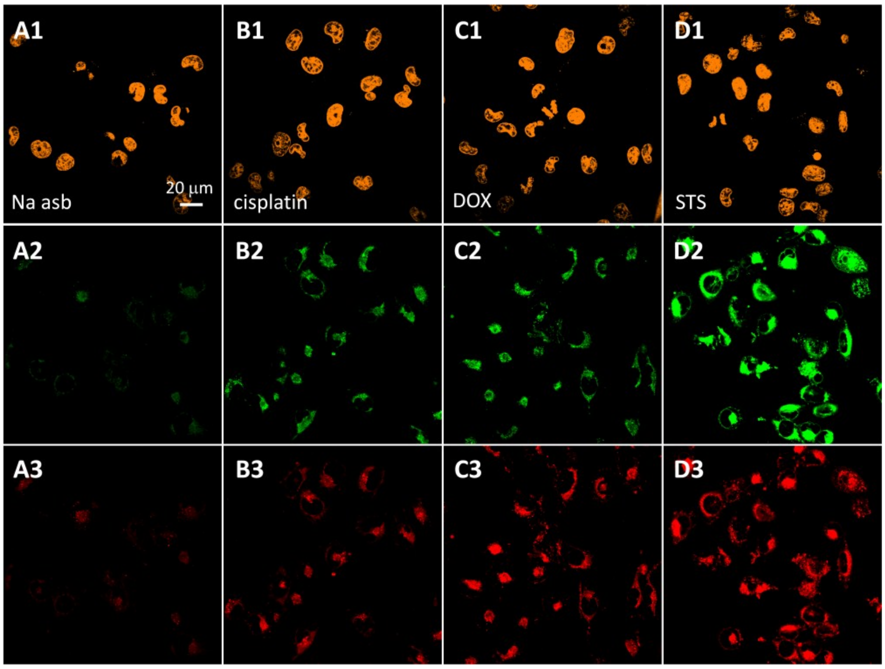

Figure S18. Confocal images of Cou-DEVD-TPETP (10 μM) incubated MDA-MB-231 cells upon treatment with (A) sodium ascorbate (Na asb), (B) cisplatin, (C) DOX and (D) STS. Orange fluorescence (nucleus dyed with SYTO® orange, A1-D1, E_x: 543 nm, E_m: 610-640 nm); green fluorescence (Cou-DEVD, A2-D2, E_x: 405 nm; E_m: 505-525 nm); red fluorescence (TPETP residue, A3-D3, E_x: 405 nm, E_m: > 650 nm).

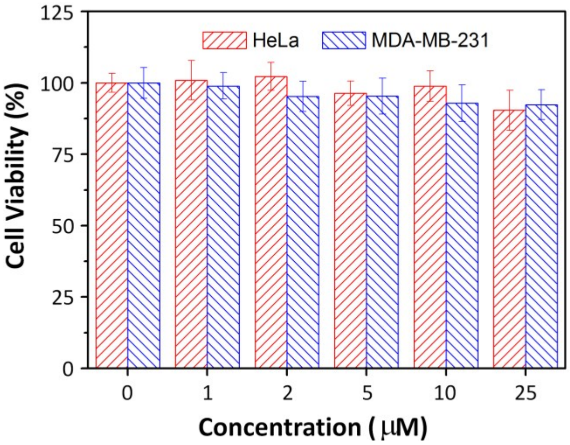

Figure S19. The viability of HeLa and MDA-MB-231 cells upon incubation with Cou-DEVD-TPETP at different concentrations for 48 h.
